# Deep sequencing reveals a novel class of bidirectional promoters associated with neuronal genes

**DOI:** 10.1186/1471-2164-15-457

**Published:** 2014-06-10

**Authors:** Hai Yang Hu, Liu He, Philipp Khaitovich

**Affiliations:** CAS Key Laboratory of Computational Biology, CAS-MPG Partner Institute for Computational Biology, 320 Yue Yang Road, 200031 Shanghai, China; Freie Universität Berlin, Kaiserswerther Str. 16-18, Berlin, 14195 Germany; Max Planck Institute for Evolutionary Anthropology, Deutscher Platz 6, Leipzig, 04103 Germany

**Keywords:** Bidirectional promoter, RNA transcriptome, lncRNA, De novo assembly, RNA sequencing, Human prefrontal cortex

## Abstract

**Background:**

Comprehensive annotation of transcripts expressed in a given tissue is a critical step towards the understanding of regulatory and functional pathways that shape the transcriptome.

**Results:**

Here, we reconstructed a cumulative transcriptome of the human prefrontal cortex (PFC) based on approximately 300 million strand-specific RNA sequence (RNA-seq) reads collected at different stages of postnatal development. We find that more than 50% of reconstructed transcripts represent novel transcriptome elements, including 8,343 novel exons and exon extensions of annotated coding genes, 11,217 novel antisense transcripts and 29,541 novel intergenic transcripts or their fragments showing canonical features of long non-coding RNAs (lncRNAs). Our analysis further led to a surprising discovery of a novel class of bidirectional promoters (NBiPs) driving divergent transcription of mRNA and novel lncRNA pairs and displaying a distinct set of sequence and epigenetic features. In contrast to known bidirectional and unidirectional promoters, NBiPs are strongly associated with genes involved in neuronal functions and regulated by neuron-associated transcription factors.

**Conclusions:**

Taken together, our results demonstrate that large portions of the human transcriptome remain uncharacterized. The distinct sequence and epigenetic features of NBiPs, as well as their specific association with neuronal genes, further suggest existence of regulatory pathways specific to the human brain.

**Electronic supplementary material:**

The online version of this article (doi:10.1186/1471-2164-15-457) contains supplementary material, which is available to authorized users.

## Background

The advent of high-throughput sequencing has ushered in a new chapter in transcriptome studies, allowing sequencing and mapping of all transcripts present in a given sample, independent of the existing genome annotation. The application of high-throughput sequencing to the characterization of human transcriptomes in different tissues and developmental stages has already revealed thousands of novel transcripts and novel transcript isoforms [1], and resulted in the recognition of long non-coding RNAs (lncRNAs) as a permanent feature of the human and mammalian transcriptome, as well as the identification of novel transcript types, such as piwi-interacting RNA and circular RNA [2–6]. Transcript annotation has been further aided by the introduction of strand-specific RNA-sequencing (RNA-seq) protocols allowing identification of sense and antisense transcripts [7, 8], as well as specific protocols designed to identify the 5′-end and 3′-end of transcripts: deepCAGE and 3P-Seq [9, 10].

Despite considerable efforts, human transcriptome annotation remains incomplete. This is largely due to the nature of the novel transcriptome elements: most of lncRNAs and other non-canonical transcripts are expressed in a highly spatial- and temporally- specific manner, *i.e.* their repertoires differ greatly among tissues, cell types and ontogenetic stages [1, 4]. Furthermore, canonical protein-coding genes have been shown to undergo alternative splicing, and use alternative transcription start and termination sites across tissues, cell types and ontogenetic stages, further contributing to transcriptome heterogeneity [11]. Among human tissues, both protein-coding and lncRNA transcripts are reported to show the greatest heterogeneity in testis and brain [12].

Correct and comprehensive identification of the transcripts expressed in a given tissue is a critical step towards reconstruction of regulatory and functional interactions. For instance, regulatory network reconstruction relies on identification of transcription factor and microRNA binding sites, which in turn require knowledge of the transcription start site position and 3′ untranslated region (UTR) boundaries in a given sample. Growing recognition of the regulatory roles played by lncRNAs, which may act as *cis*- or *trans*- regulators of other transcripts, further highlights the need for complete characterization of the transcriptome as a prerequisite for regulatory and functional network reconstruction. Many lncRNAs are located in the proximity of protein-coding genes, and are transcribed from certain types of regulatory regions, further indicating their regulatory potential. lncRNAs transcribed from enhancer regions upon cellular membrane depolarization (eRNA) have been linked to the elevated expression of neighboring genes in murine neural cells [13]. Similarly, diverse populations of lncRNAs have been shown to originate from known promoter regions [14]. In human and murine embryonic stem cells (ESCs), more than half of all expressed lncRNAs represented divergent transcription from bidirectional promoters of known protein-coding genes [15]. While in ESCs, these divergent lncRNAs were associated with elevated expression of the corresponding protein-coding genes, other studies have reported negative regulation of protein-coding genes by divergent lncRNA expression [16].

In this study we took advantage of a large strand-specific RNA-seq dataset to characterize the transcriptome of one of the most heterogeneous and complex human tissues – human prefrontal cortex (PFC). Our results demonstrate that systematic transcriptome characterization not only reveals thousands of yet unannotated transcripts, but also allowed us to discover a novel type of bidirectional promoters comprised of canonical protein-coding gene and tissue-specific novel non-coding transcript pairs. Most remarkably, these bidirectional promoters represent a specific promoter category, characterized by its own sequence and epigenetic signature and specifically associated with expression of neuronal genes.

## Results

### More than 40% of transcripts expressed in the human brain are novel

**Figure 1:**
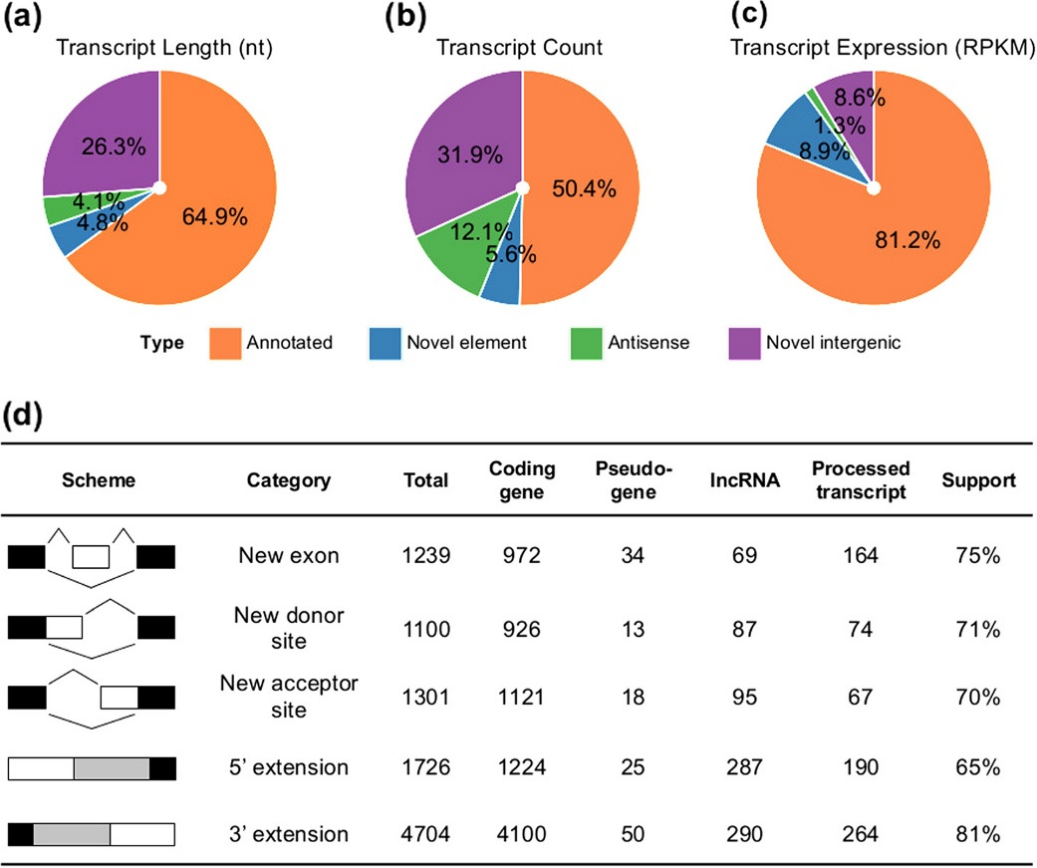
**Annotated and novel portions of the human PFC transcriptome. (a)**, **(b)**, **(c)** The proportion of four transcript types – annotated transcripts (orange), novel elements of annotated transcripts (blue), antisense transcripts (green), and novel intergenic transcripts (purple) – with respect to the total transcriptome length, transcript count and expression level, respectively. **(d)** Categories of novel elements of annotated transcripts detected in the human PFC transcriptome. Black and grey boxes indicate annotated exons and UTRs, white boxes represent novel transcript elements. The “Support” column shows the percentage of novel transcript isoforms confirmed by Oases and Cufflinks transcriptome assembly algorithms, and additionally supported by H3K4me3 modification peaks and transcript polyA tails (see Methods).

To explore the dynamics of the human prefrontal cortex polyA-plus transcriptome, we took advantage of strand-specific high-throughput sequencing data collected in the prefrontal cortex (PFC) of 14 human individuals with an age range from 2 days to 98 years [17] (Additional file 1: Table S1). These data contained an average of 21 million 100-nt-long reads per sample, with a total of 296 million reads (Additional file 2: Table S2). To avoid limitations imposed by transcriptome read mapping to the genome, we first assembled transcripts *de novo* using the Trinity algorithm [18]. Of the raw sequence reads, 96% were retained after quality control and subsequently used in the transcript assembly. The assembly resulted in 332,993 transcript contigs with an average length of 1,005 nt and minimum length set to 300 nt. Of them, 307,543 (92.4%) could be unambiguously and uniquely aligned to the human reference genome using GMAP [19]. Merging contigs that overlapped with each other on the human genome resulted in 92,705 contig clusters. The total length of these assembled transcripts was 94,989,683 nt. Of them, 61,650,777 nt (64.9%) overlapped with human annotated transcripts (Ensembl gene annotation, version 64 [20]) covering 61% of all annotated exons, while the remaining 36,938,906 nt (35.1%) represented as yet unannotated portions of the human brain transcriptome. Among the unannotated transcripts 4,124,023 nt (4.2%) originated from novel elements of annotated genes, such as novel exons and novel exon extensions; 3,877,147 nt (3.6%) from the antisense strand of annotated genes; and 28,937,736 nt (29.7%) from novel intergenic transcripts (Figure 1a). Accordingly, of the 92,705 assembled contig clusters, 51,948 (56%) overlapped with at least one annotated transcript, while the remaining 40,758 (44%) originated from gene antisense and intergenic regions (Figure 1b). In terms of transcriptome read counts, and reflecting the expression level of the transcripts, annotated transcripts accounted for 81% of all transcriptome reads, novel elements of annotated genes and intergenic transcripts – for 9% each, and antisense transcripts – for the remaining 1% (Figure 1c).

Notably, our analysis also revealed potential gaps, not only in the human genome annotation, but also in the human genome itself. We found 368 human transcript contigs that could not be mapped to the reference human genome (hg19), but could be aligned unambiguously to at least one of the following non-human reference genomes: chimpanzee, orangutan, macaque or mouse genomes. Cumulatively these contigs cover 146,035 bases and include 12 putative protein-coding genes and 101 putative novel exons from another 10 annotated protein-coding genes.

### Annotated human transcripts contain numerous novel elements

Among the 51,948 assembled contig clusters that were located within annotated transcripts, 3,699 clusters, composed of 12,822 contigs, contained transcript elements not covered by the existing annotation. These elements included: 972 novel internal exons located in 754 protein-coding genes; 926 and 1,121 novel donor and acceptor splice sites containing canonical splicing signals located in 1,687 protein-coding genes; as well as 1,224 and 4,100 novel 5′UTR and 3′UTR extensions with a length of at least 100 nt and located in 1,952 protein-coding genes. 9.2% of these novel transcript elements were highly expressed (top 25% quantile of the annotated protein-coding transcripts in the corresponding gene), while 35.8% were moderately expressed (within the 75% quantile of the annotated protein-coding transcripts in the corresponding gene). Besides protein-coding genes, 267 novel exons, 354 splice boundaries and 1,106 5′/3′UTR extensions were found in annotated pseudogenes, lncRNAs and processed transcripts from 1,531 contig clusters (Figure 1d). 75.6% of these novel elements could be validated by the other transcript assembly algorithms, Oases [21] or Cufflinks [22], as well as by the presence of H3K4me3 modification peaks, commonly associated with active promoters, or sequence reads corresponding to transcripts’ polyA tails (see Additional file 3: Supplementary Methods).

### Novel transcripts show properties of long non-coding RNA

**Figure 2:**
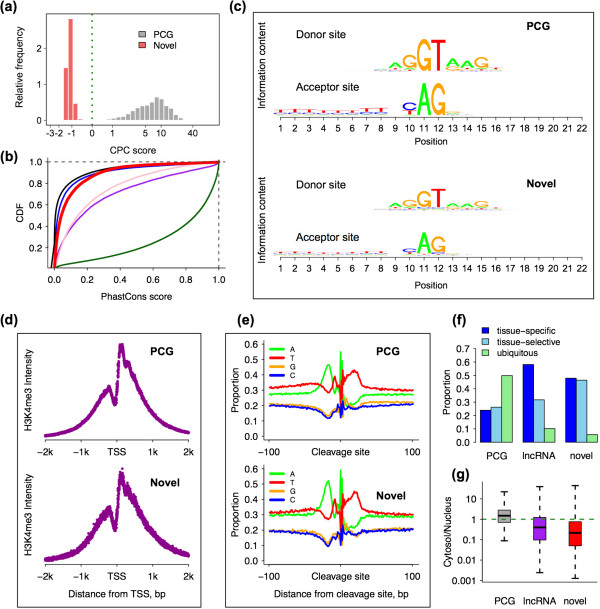
**Properties of novel transcripts. (a)** Distributions of coding potential scores estimated for novel transcripts (red) and annotated protein-coding genes (PCG, gray) using CPC (Coding Potential Calculator). Negative scores indicate low coding potential. **(b)** Cumulative distribution of exon sequence conservation levels estimated using PhastCons scores based on 17 vertebrate species’ genomes. The colors indicate novel transcripts (red), random intergenic regions (black), annotated lncRNAs (blue), pseudogenes (pink), UTR exons (purple) and protein-coding exons (green). **(c)** Nucleotide composition at and around the splice sites (positions 11-12) of annotated protein-coding genes (PCG, upper panel) and novel transcripts (bottom panel). **(d)** H3K4me3 modification profiles at the promoter region of annotated protein-coding genes (PCG, upper panel) and novel transcripts (bottom panel). Transcription start site (TSS) position of novel transcripts was estimated using deepCAGE data from brain tissues. **(e)** Nucleotide composition around the transcript termination sites (TTS) of annotated protein-coding genes (PCG, upper panel) and novel transcripts (bottom panel). **(f)** Tissue specificity of expression for annotated protein-coding genes (PCG), annotated lncRNA (lncRNA) and novel transcripts (novel), calculated using Body Map data. **(g)** Cellular localization (cytosol to nucleus expression level [RPKM] ratio) of annotated protein-coding genes (PCG), annotated lncRNA (lncRNA) and novel transcripts (novel).

Among the 92,705 contig clusters identified in our data, 40,758 had no overlap with genome annotation (Ensembl version 64) (Additional file 4: Supplementary data 1). Some of these transcripts showed a moderate expression: using expression of protein-coding genes as a reference, 3.1% of contig clusters were highly expressed (top 25% quantile of all annotated protein-coding transcripts) and 26.2% - moderately expressed (within the 75% quantile of all annotated protein-coding transcripts). Based on a coding potential estimation using the CPC algorithm [23], 99% of these transcripts have negative coding potential score and, therefore, may represent novel long non-coding RNAs (lncRNAs) or novel lncRNA fragments (Figure 2a). Besides negative coding potential scores, novel contigs clusters displayed other features characteristic of annotated lncRNAs. Specifically, contig clusters containing multiple exons displayed canonical donor (68%) and acceptor (64%) splice sites (Figure 2c). Further, 30% of the novel contigs clusters featured H3K4me3 modification peaks within 2 kb region from their 5′end (Figure 2d, simulations, *p* < 0.01, Additional file 3: Supplementary Methods). Consistent with the polyA enrichment procedure used during sequencing library preparation, 35% of all novel contigs clusters contained detectable polyA tails within a 2 kb region from their 3′end (Figure 2e, simulations, *p* < 0.01, Supplementary Methods). The novel contig clusters identified in our study were significantly more conserved at the DNA sequence level among 17 vertebrate species when compared to randomly selected intergenic regions or annotated human lncRNAs (Kolmogorov–Smirnov test, *p* < 0.0001) (Figure 2b). In agreement with previous studies reporting a high tissue-specificity for lncRNA expression [4], among the 31,006 novel contig clusters that could be quantified in the Human Body map data (mean expression > 0.1 RPKM across tissues), 89% were expressed in a tissue-specific (38%) or tissue-selective manner (51%) (Figure 2f). Furthermore, similar to known lncRNAs, novel transcripts were preferentially localized in the nucleus (Figure 2g). Taken together, these features indicate that identified contig clusters may, in many cases, represent as yet unannotated human lncRNAs or lncRNA fragments.

The RNA-seq data we used to identify novel transcripts represents a human PFC developmental time-series. Accordingly, 20% of the transcript clusters representing novel lncRNAs showed significant expression level change with age (polynomial regression, permutation *p* < 0.01, *q* < 0.02 see Methods). Notably, the majority of these transcripts were highly expressed in early development (Fisher’s exact test, *p* < 0.001 after Bonferroni correction, Additional file 5: Figure S1 and Additional file 6: Table S3).

### Properties of antisense transcripts

**Figure 3:**
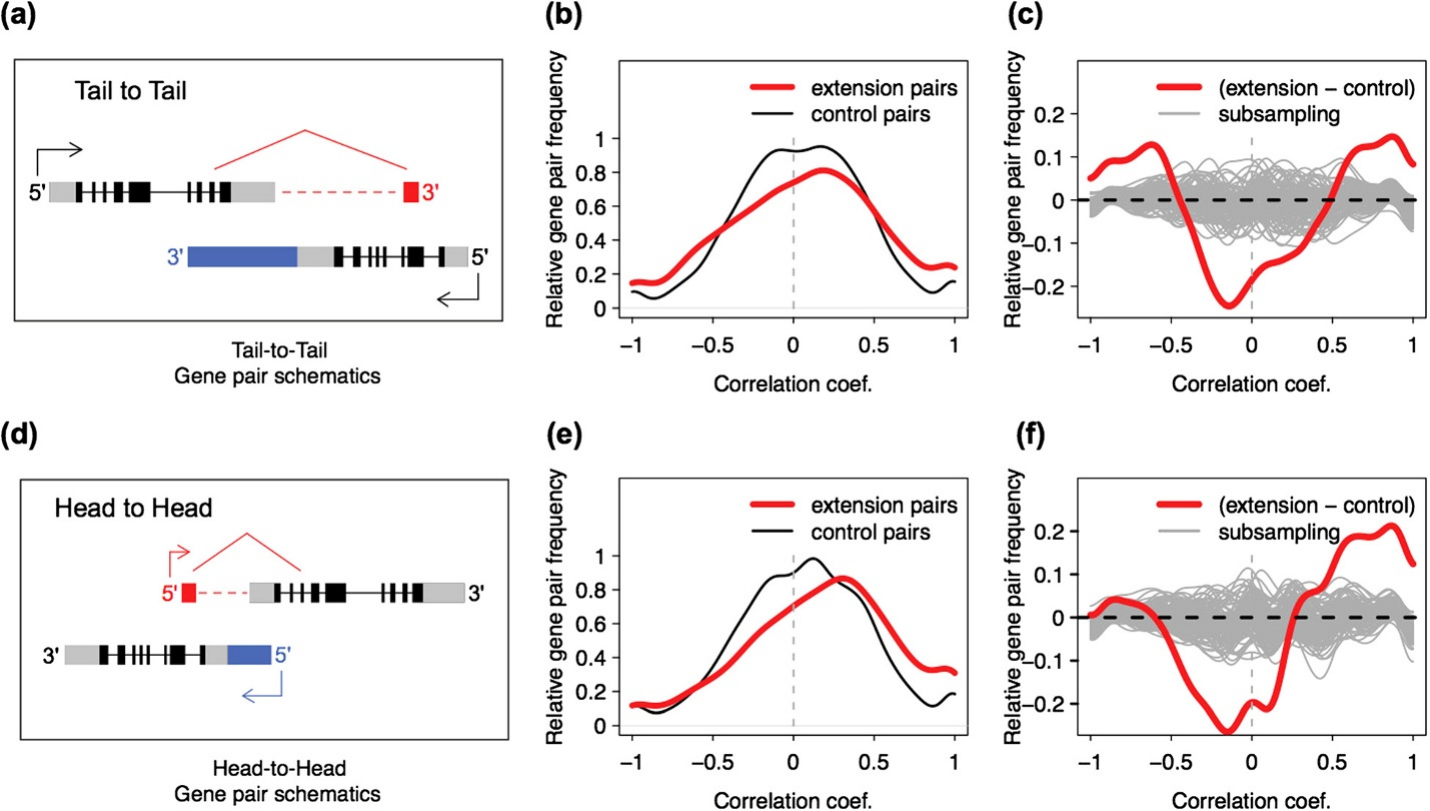
**Relationship between sense and antisense transcript expression. (a)**, **(d)** Schematic representations of tail-to-tail and head-to-head overlapping gene pairs. Black and gray boxes represent annotated protein-coding and untranslated exons, red and blue boxes indicates novel antisense transcript elements. **(b)**, **(e)** Distribution of Pearson correlation coefficients calculated based on expression of sense and antisense transcripts in tail-to-tail (panel **b**) or head-to-head (panel **e**) gene pairs (red), and control non-overlapping gene pairs (black), during postnatal PFC development. **(c)**, **(f)** Difference between the kernel density distribution of the overlapping tail-to-tail (panel **c**) or head-to-head (panel **f**) gene pairs’ correlation and the control ones. Red line indicates the overlapping pairs, while the gray lines represent 100 simulation results, generated by randomly subsampling the same number of control pairs as overlapping pairs.

Use of a strand-specific sequencing protocol allowed us to evaluate the expression of transcripts originating from the antisense strand of annotated protein-coding gene regions. Among the 92,705 contig clusters assembled in this study, 13,218 were located on the antisense strand relative to annotated protein-coding gene regions. Of these, 11,217 were completely unannotated and 2,001 overlapped partially with annotated genes located on the same strand. Taken together, these antisense contig clusters resulted in 1479 annotated and 870 novel sense/antisense overlapping coding gene pairs (see Methods, Figure 3a and d, Additional file 7: Table S4).

One notable feature of the novel antisense contig clusters was their distributions within antisense regions: while annotated antisense transcripts tend to cluster in the 5′ and 3′ region of the sense gene, the novel antisense contig clusters were distributed much more uniformly (Additional file 5: Figure S2). To test whether the novel antisense contig clusters may represent long extensions of annotated transcripts located nearby, we searched for reads corresponding to splicing junctions connecting antisense contig clusters and neighboring genes in our RNA-seq data (Additional file 3: Supplementary Methods). Indeed, we identified 185 such connections, 136 of them representing 3′ extensions (tail-to-tail gene pairs) and 49 representing 5′ extension (head-to-head gene pairs), while only 16 would be expected by chance (simulation test, *p* < 0.001, Additional file 7: Table S4). Notably, 3′ extensions verified by splice junctions were also distributed broadly within antisense regions, with the longest reaching the 5′-end of the sense gene.

What is the influence of these antisense transcripts on gene expression of sense/antisense gene pairs? Previous studies have indicated that the majority of identified sense/antisense gene pairs are positively correlated, while inversely correlated pairs also exist [8, 24–26]. To test the effect of antisense transcription on expression of the sense genes we took advantage of the age-related changes in expression of sense and antisense transcripts during human brain development, which could be documented in our dataset. In agreement with previous studies, we observed a significant excess of both positive and negative correlation for 1,330 tail-to-tail annotated and novel sense/antisense gene pairs compared to equidistant non-overlapping gene pairs (Figure 3b and c, Additional file 5: Figure S3, Additional file 7: Table S4). For 1,152 sense/antisense gene pairs with overlapping 5′ regions (head-to-head gene pairs), only an excess of positive correlations was observed (Figure 3e and f, Additional file 5: Figure S4, Additional file 7: Table S4). Positively correlated expression of head-to-head gene pairs may reflect shared open chromatin structure and regulation. More interestingly, the observations of positively and inversely correlated expression patterns from tail-to-tail pairs appears to represent a more complicated regulatory phenomenon that includes agonistic interactions between sense and antisense transcription, such as the previously proposed spatial collision of transcription and splicing machineries [27–30].

### Novel upstream antisense lncRNAs expressed in the PFC originate from a new class of bidirectional promoters

**Figure 4:**
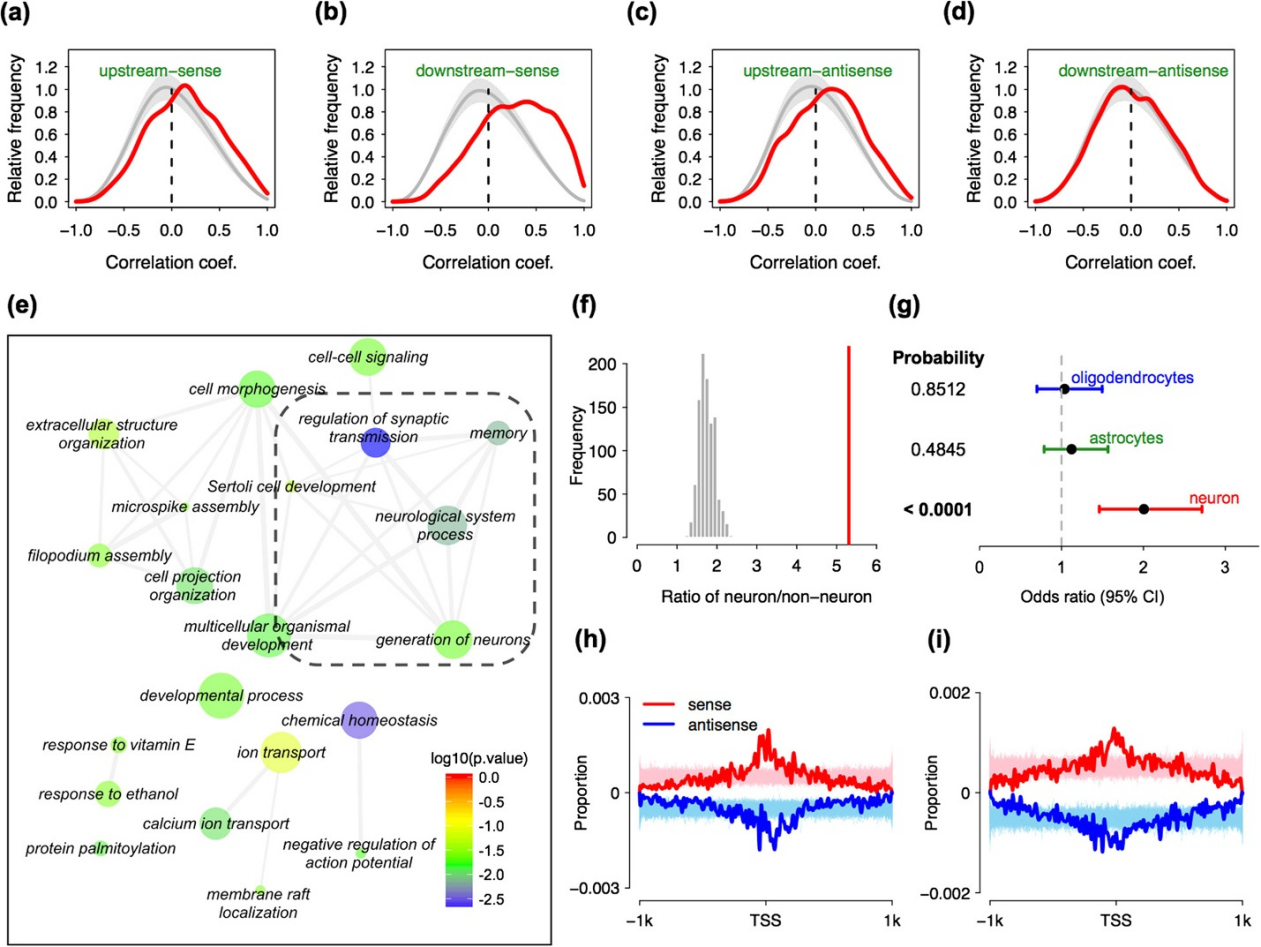
**Properties of genes associated with novel upstream-antisense** ***lncRNAs.*** **(a)**, **(b)**, **(c)**, **(d)** Distribution of Pearson correlation coefficients calculated based on the expression of protein-coding genes and nearest novel lncRNAs (red curve). The gray curves show the average correlation coefficient distribution based on 200 permutations of neighboring novel lncRNAs and protein-coding gene relationships. The gray shaded areas show standard error of the curve estimates. The distributions are shown for four possible genomic configurations of lncRNA relative to protein-protein-coding genes: upstream-sense, downstream sense, upstream-antisense and downstream-antisense. **(e)** GO terms enriched in 273 protein-coding genes associated with upstream-antisense novel lncRNAs. The node color indicates the GO term’s enrichment p-value, the node size is proportional to the GO term’s annotated gene number. Dashed rectangle indicates GO terms associated with neuronal functions. **(f)** Expression specificity of 273 protein-coding genes associated with upstream-antisense novel lncRNAs, calculated based on H3K4me3 modification from neurons and non-neural cells of human PFC. The red bar shows neuron/non-neuron cells ratio for the 273 genes, the gray bars represent the ratio distribution calculated by 1,000 permutations of 273 randomly selected expressed genes. **(g)** Expression specificity of 273 protein-coding genes associated with upstream-antisense novel lncRNAs calculated based on cell type specific expression data from mouse neocortex. The bars show Fisher’s test odds ratio with 95% confidence interval for enrichment of the 273 genes among mouse orthologs preferentially expressed in oligodentrocytes (blue), astrocytes (green) and neurons (red). The numbers show Fisher’s test p-values. **(h)**, **(i)** The TFBS density profiles of two enriched TFs, ETF (panel **h**) and ZF5 (panel **i**), within NBiPs. The red and blue curves show observed TFBS density distributions at sense and antisense strands. The pink and the light blue curves represent TFBS density distributions calculated by 1,000 permutations of TFBS prediction across dinucleotide shuffled NBiP sequences.

Previous studies have shown that the majority of the novel transcripts located outside of annotated gene regions, both sense and antisense, may represent as yet unannotated extensions of known genes [31]. Indeed, among 39,364 novel contig clusters with expression greater than 0.1 RPKM, 14,235 (36.2%) were located within 4 kb from annotated transcript boundaries (simulations, *p* < 0.04, Supplementary data 1). Based on the DNA strand, and relative position with respect to the nearest annotated transcript region, these 14,235 novel transcripts could be further classified into four categories: upstream-sense (1,323 or 9.3%), downstream-sense (6,965 or 48.9%), upstream-antisense (2,964 or 20.7%), and downstream-antisense (2,983 or 21.1%). We found a significant excess of positive correlations between the expression of transcripts represented by novel contig clusters and the expression of nearby protein-coding genes for the upstream-sense, downstream-sense and upstream-antisense categories (Figure 4a, b and c). No significant correlation signal was found for the downstream-antisense category (Figure 4d).

While novel contig clusters located on the sense strand may represent 5′ and 3′ extensions of known genes, transcripts originating from the antisense strand must have an independent origin. Indeed, there is no correlation between the expression of annotated genes and nearby antisense transcripts located downstream. By contrast, a significant excess of positive correlations between annotated genes and upstream-antisense transcripts may indicate shared regulation, presumably at as yet unannotated bidirectional promoters. Indeed, a signature of divergent transcription characteristic of bidirectional promotes can be observed for the upstream-antisense transcripts and the corresponding annotated genes, using public human brain deepCAGE tag data from FANTOM4 [9]. The divergent transcription was strong for all upstream-antisense novel transcript/gene pairs, and particularly pronounced for the 273 upstream-antisense novel transcript/gene pairs that showed a significant positive correlation in the PFC time-series data (Pearson correlation, *p* < 0.05 after Benjamini-Hochberg correction), compared to other promoters annotated as unidirectional (Fisher’s exact test, *p* < 0.0001, Additional file 5: Figure S5, Additional file 8: Table S5, Additional file 4: Supplementary data 1).

### Novel upstream antisense lncRNAs expressed in PFC are linked to neural function

Functional analysis of the protein-coding genes associated with the 273 novel upstream-antisense transcripts revealed a strong and significant enrichment in 21 Gene Ontology (GO) functional terms after redundancy reduction [32], including neuronal functions such as “memory”, “generation of neurons” and “regulation of synaptic transmission” (hypergeometric test, *p* < 0.05 after Benjamini-Hochberg correction, Figure 4e, Additional file 9: Table S6). Consistently, the 273 genes are preferentially expressed in neurons, as gauged from H3K4me3 modification data collected in neurons and non-neuronal cells in the human PFC [33] (Simulation test, *p* < 0.0001, Figure 4f), and neuron-specific gene expression data collected in the mouse brain [34] (Fisher’s exact test, *p* < 0.0001 after Bonferroni correction, Figure 4g, see Methods). By contrast, protein-coding genes associated with novel transcripts from the other three categories did not show any significant functional enrichment. More surprisingly, known bidirectional promoters (KBiPs), either consisting of two protein-coding genes, or protein-coding gene and known lncRNA pairs, expressed in the human PFC showed no significant enrichment in neural functions. Instead, these genes were significantly underrepresented in neuronal functions, but overrepresented in biological processes related to RNA processing, DNA repair, DNA metabolic process, and ribonucleoprotein complex biogenesis (Additional file 10: Table S7). Similarly, annotated genes transcribed from annotated unidirectional promoters (UniPs), and showing no evidence of upstream antisense expression in our data, were not enriched in neuronal functions, but instead in biological processes related to signal transducer activity and receptor activity (Additional file 11: Table S8). Thus, the bidirectional promoters identified in our study (novel bidirectional promoters or NBiPs) may represent a separate promoter category that differs from both UniPs and KBiPs and particular to genes expressed in neurons and/or associated with neuronal functions.

### Novel bidirectional promoters are enriched in transcription factors associated with neurons

The unique functional features of NBiPs prompted us to explore transcription factors that may regulate this promoter type. Several transcription factors that are preferentially associated with bidirectional promoters have been identified by previous studies [35, 36]. Comparing transcription factor binding site (TFBSs) density within 2 kb of NBiPs and KBiPs revealed 10 TFBSs that correspond to 11 transcription factors (TFs) enriched in NBiPs, and 6 TFBSs corresponding to 8 TFs enriched in KBiPs (Fisher’s exact test, *p* < 0.05 after Benjamini-Hochberg correction & odds ratio > 1.3, Additional file 12: Table S9 and Additional file 13: Table S10). The association between enriched TFs and NBiPs was further confirmed for five TFs by the significant correlation of their expression profiles and the expression profiles of the predicted target transcripts originating from the NBiPs (permutations, *p* < 0.05, Additional file 12: Table S9). Furthermore, for two of the five enriched and correlated TFs, significant peaks of TFBS density profiles were detected in the center of the NBiP regions (Figure 4h,i). Notably, with respect to function, TFs enriched in NBiPs were significantly co-cited with the terms “neural” or “neuron” (CoCiter [37], *p* < 0.01, Additional file 12: Table S9). By contrast, TFs enriched in KBiPs showed no such association (CoCiter, *p* > 0.2, Additional file 13: Table S10). Thus, NBiPs may represent an integral part of a regulatory mechanism specific to a set of neuronal genes and involving specific neuron-related TFs.

### Novel bidirectional promoters show unique DNA sequence and epigenetic features

**Figure 5:**
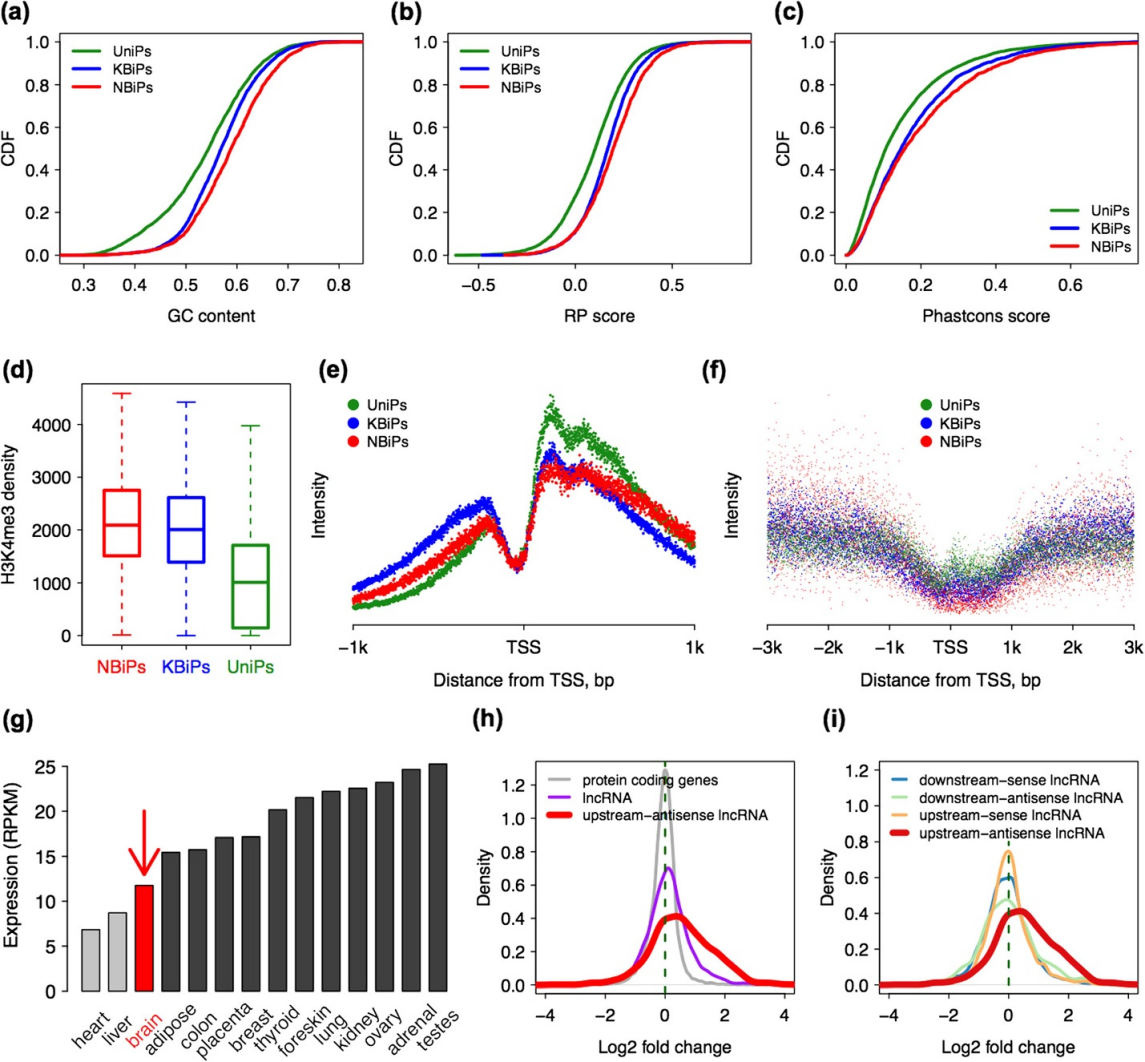
**Sequence and epigenetic features of different promoter types. (a)**, **(b)** and **(c)** The cumulative distributions of GC content, Regulatory Potential and sequence conservation for the three promoter types: UniPs (green), KBiPs (blue) and NBiPs (red). All measurements are based on a 2 kb region surrounding the TSS. Promoter sequence conservation was calculated using PhastCons scores, based on 17 vertebrate species’ genomes. Promoter Regulatory Potential was calculated using Regulatory Potential (RP) scores (see Methods). **(d)**, **(e)** The density (panel **d**) and the shape (panel **e**) of H3K4me3 modification profiles at each of the three promoter types. **(f)** DNA methylation profile at each of the three promoter types. **(g)** PABPN1 expression across human tissues calculated using Body Map data. **(h)**, **(i)** The expression change distribution for different transcript types in a PABPN1 knockdown experiment. The positive values indicate expression upregulation following PABPN1 knockdown.

The unique functional and regulatory features of NBiPs might suggest a specific sequence and epigenetic signature for this promoter type. Indeed, compared with UniPs and KBiPs, NBiPs show significant differences with respect to all common sequence and epigenetic features: GC content, regulatory potential, sequence conservation, H3K4me3 modification profile, and DNA methylation status. Specifically, NBiPs have a higher GC content and higher regulatory potential, measured as a Regulatory Potential (RP) Score [38], than both UniPs and KBiPs (Kolmogorov-Smirnov test, *p* < 0.0001; Figure 5a,b). Further, NBiPs are more conserved at the DNA sequence level than KBiPs (Kolmogorov–Smirnov test, *p* < 0.001), while both types of bidirectional promoters are more conserved than UniPs (Kolmogorov-Smirnov test, *p* < 0.0001, Figure 5c). H3K4me3 modification density, measured in the human PFC neurons [33], is higher at NBiPs than KBiPs indicating promoter activity (Wilcoxon test, *p* < 0.001). Further, H3K4me3 modification density was greater at both types of bidirectional promoters compared to UniPs (Wilcoxon test, *p* < 0.0001; Figure 5d). Notably, besides the overall H3K4me3 modification density differences, the shape of H3K4me3 modification profiles differs among the three promoter types (Figure 5e). Specifically, UniPs show starkly asymmetric H3K4me3 modification profiles with much of the modification density located downstream of the protein-coding gene transcriptional start site (TSS). By contrast, the shape of H3K4me3 modification profile is more symmetric relative to the TSS for both NBiPs and KBiPs, with the most symmetric signatures observed at KBiPs. This difference in H3K4me3 modification signature could be reproduced using other H3K4me3 modification datasets obtained from human and rhesus macaque PFC samples, as well as HeLa cells [39–41] (Additional file 5: Figure S6). By contrast, the input control showed no significant differences in shape and density for H3K4me3 modification profiles among the three promoter types (Additional file 5: Figure S7). Lastly, DNA methylation levels measured in the human PFC [39] also differed among the three promoter types: DNA methylation levels are high at UniPs, intermediate at KBiPs and the lowest at NBiPs (Wilcoxon test, *p* < 0.0001; Figure 5f). KBiPs are comprised of two types of bidirectional promoters: one formed by two protein-coding genes (pcKBips, *n* = 806), and the other – by a protein-coding gene and known lncRNA pair (lncKBips, *n* = 359). Do the aforementioned sequence and epigenetic features characteristic of NBiPs also distinguish them from bidirectional promoters containing known lncRNAs (lncKBips)? To answer this, we directly compared the DNA sequence composition and epigenetic features of NBiPs and lncKBiPs. Except for the shape of H3K4me3 modification profile, there are significant differences between other features for these two promoter types. Furthermore, the sequence and epigenetic properties of lncKBiPs closely resembled those of known bidirectional promoters formed by pairs of protein-coding genes (pcKBiPs). Besides promoter features, the effect of PABPN1 knockdown on lncRNA that are associated with NBiPs differed from the effect seen for known lncRNA and protein-coding genes associated with lncKBiPs and pcKBiPs (Additional file 5: Figure S8). Thus, in the brain, NBiPs formed by lncRNA represent a distinct type of bidirectional promoter with characteristic structural and regulatory properties when compared to known bidirectional promoters, including those containing known lncRNA.

## Discussion

Knowledge of the composition of the human prefrontal cortex transcriptome is critical for studying the complexity of RNA transcription and regulation, as well as its impact on neuronal functions. Here, by applying a strand-specific RNA sequencing procedure to different stages of postnatal development, we have obtained one of the most complete and dynamic pictures of the human prefrontal cortex transcriptome. Several interesting observations have emerged.

First, despite the substantial efforts made towards human brain transcriptome characterization in previous decades, more than 50% of PFC transcripts reconstructed in our study represent novel transcriptome elements. These elements include novel exons and exon extensions of annotated protein-coding and lncRNA genes, antisense transcripts and novel lncRNAs. One potentially interesting feature of antisense transcripts highlighted by our study is their length. While many of the antisense transcripts found in our study represent as yet unannotated extensions of the neighboring genes, these extensions frequently stretch for hundreds of base pairs, traversing the entire length of the sense genes. In agreement with previous studies, these antisense transcripts can display both negative and positive correlation with expression of the sense genes [25].

Second, while most of the lncRNAs expressed in the prefrontal cortex (39.8%) localize in close proximity ((<4 kb)) to known protein-coding genes, one fraction of these transcripts, the lncRNAs located upstream of the protein-coding genes on the antisense strand, particularly stands out. Specifically, these transcripts: (a) show a significantly positive correlation with the expression of the upstream protein-coding genes; (b) originate from a specific class of bidirectional promoters showing unique sequence and epigenetic features; (c) are highly enriched upstream of genes that are expressed in neurons and involved in neuronal functions; and (d) are enriched in TFs shown to be linked to neurons.

Bidirectional promoters are a common feature of the human genome, and have also been described in the mouse and other species [42, 43]. In humans, 10% of protein-coding genes were annotated to originate from bidirectional promoters [43]. Remarkably, genes preferentially expressed in brain and involved in neural functions were depleted at these known bidirectional promoters [44]. This result was further confirmed in this present study. By contrast, novel bidirectional promoters showing divergent transcription of novel and potentially brain-specific lncRNAs, are highly enriched in neuronal genes. The novel bidirectional promoters identified in our study are also distinct from both known bidirectional promoters and unidirectional promoters with respect to many aspects of sequence composition and epigenetic features, including H3K4me3 chromatin modifications and DNA methylation. Thus, they may represent a novel promoter type specifically associated with the expression of neuronal genes and regulated by a specific set of TFs. Intriguingly, TFs showing significant association with this promoter type, include all three methylation resistant TFs (AP-2 family, EGR family and ZF5) representing three of the top four discriminatory features used to predict methylation status of CpG islands in the human brain [45]. This fact may explain the unique DNA methylation signature of the NBiPs observed in our study.

Expression of lncRNAs from bidirectional promoters has been previously shown in many human cell types, including human embryonic stem cells (hESCs) where >60% promoters might be bidirectional and associated with divergent lncRNAs [15]. Notably, even though we find no significant overlap between bidirectional promoters described in hESCs and NBiPs identified in this study, in both cases expression of protein-coding genes correlated positively with expression of divergent lncRNAs. It is, however, unclear whether this positive correlation represents a regulatory effect of lncRNAs, or a passive consequence of the transcriptional activation of the divergent protein-coding genes. Most human promoters bind polymerase complexes in a bidirectional manner and are therefore capable of initiating transcription in both directions [42]. Thus, we cannot exclude that the presence of lncRNAs at the novel type of bidirectional promoters identified in our study may represent a passive byproduct of neuronal gene transcription from this specific promoter type.

Transcripts expressed in the PFC, and more generally in the brain, are characterized by extended 3′UTR regions [12]. This phenomenon may in part be explained by the low expression of PABPN1, a gene recently shown to play a role in transcript processing [46, 47] in brain tissue (Figure 5g). Intriguingly, by reanalyzing data from [48], we found that the expression of novel lncRNAs originating from NBiPs was starkly increased in a PABPN1 knockdown experiment. Furthermore, this expression increase was significantly greater than for other lncRNA types (Figure 5h and i). This indicates that the strong expression of lncRNAs originating from NBiPs in the human PFC could be due to this general transcript processing mechanism.

## Conclusions

Taken together, our results demonstrate that large portions of the human transcriptome remain uncharacterized and even unknown. We further show that more detailed transcriptome characterization may lead to the identification of new types of regulatory elements, such as a novel class of bidirectional promoters associated with the expression of neuronal genes. Finally, our study confirms pervasive transcription of lncRNAs in the human PFC, again raising the question of their functionality.

## Methods

### Quality evaluation of strand-specific sequencing

The RNA-Seq data from 14 human individuals with an age range from 2 days to 98 years were downloaded from [17]. To evaluate the correctness of strand specificity of this dataset, total reads were mapped to the human genome (hg19) using PalMapper [49] (Additional file 2: Table S2, Additional file 3: Supplementary methods). Read distribution along sense/antisense strands was calculated based on protein-coding genes (PCG) defined by Ensembl gene annotation (version 64) (Additional file 14: Table S11). Note that within the Ensembl annotation, a substantial number of genes overlap, either on the same strand or on different strands. To avoid erroneous counting of sense and antisense reads, reads from overlapped regions on the same strand were counted only once, while reads from overlapped regions located on different strands were excluded.

Strand-specific quality evaluation was done by: (1) checking the expression correlation of protein-coding genes between two strands within each sample; (2) examining the sense/antisense ratio of the exon-spanning junction reads that had built-in directionalities (Additional file 5: Figure S9, Additional file 15: Table S12 and Additional file 3: Supplementary Methods).

### De novo transcript assembly

The quality of raw deep sequencing reads was first assessed using the FASTX tool kit (http://hannonlab.cshl.edu/fastx_toolkit/index.html). After removing low quality reads (phred score < 20), raw reads from 14 human prefrontal cortex samples were combined, resulting in a total of 284 million 100 nt strand-specific reads. These reads were used as the input data for Trinity *de novo* assembly. Trinity (version r2011-11-26) was downloaded from the Trinity homepage [18]. The assembly parameters were chosen as follows: (--seqType fq –single --CPU 80 --min_contig_length 150 --SS_lib_type F –bflyHeapSpace 260G). After removing the transcript contigs with a length shorter than 300 nt, Trinity finally reported 332,993 transcript contigs with an average length of 1,005 nt and a minimum length of 300 nt. Besides Trinity, the Cufflinks [22] and Oases [21] assembly algorithms were applied to the same human PFC RNA-seq data for the reference-based transcript assembly and reference-free *de novo* transcript assembly, respectively. A detailed description of this procedure is listed in Additional file 3: Supplementary Methods.

### Transcript contigs mapping

The transcript contigs produced by Trinity were mapped to the human genome (hg19) using GMAP [19] (version 2011-10-07) with the following parameters: (-A microexon-spliceprob 0.95 -f 1). Unambiguously and uniquely aligned transcript contigs were further required to meet the minimal identity cutoff >0.95, and the coverage cutoff >0.95. After merging overlapping mapped contigs, 92,705 separate contig clusters remained. The “known” and “novel” contig clusters classification was based on Ensembl gene annotation (version 64) [20]: assembled contig clusters that overlapped with at least one annotated transcript by at least one nucleotide were classified as “known”, the remaining contig clusters were classified as “novel”.

To identify novel contig clusters that were missing because of the incompleteness of the current human genome (hg19), we first collected contig clusters that could not be mapped to the human genome, using a relaxed mapping cutoff (mapping minimal identity >0.8, coverage >0.5), and further mapped them to four non-human genomes (chimpanzee, orangutan, rhesus macaque and mouse) using GMAP with an additional parameter (--cross-species). This resulted in 368 transcript contigs that could be aligned to at least one non-human genome (minimal identify >0.8, coverage >0.8), covering a total length of 146,035 nt. Putative protein-coding genes and exons were obtained by overlapping the 368 transcript contigs with annotations from the four non-human genomes.

### Novel elements of annotated transcripts

Novel transcribed elements of annotated genes, including novel internal exons, novel splicing donor & acceptor splicing sites and novel 5′UTR & 3′UTR extensions, were defined based on the assembled contig clusters overlapping with at least one transcript, annotated by the Ensembl database (version 64). Novel internal exons were defined based on the assembled contig clusters sharing at least one exon of annotated transcripts, and were further required to fully reside within the intron region of this annotated transcript. Novel donor and acceptor splice sites were required to share one boundary with an internal exon of an annotated transcript and containing the canonical donor/acceptor splicing sequence (GT-AG) at the novel splice boundary. Novel 5′UTR & 3′UTR extensions were required to share at least one exon with annotated transcripts and each extended region was at least 100 nt long.

The expression levels of novel and known elements of annotated transcripts was estimated using RSEM: a software package for estimating gene and isoform expression levels from RNA-Seq data with the EM algorithm [4].

### Novel elements validation

Novel internal exons, as well as novel donor and acceptor splice sites, were further validated using other transcript assembly algorithms: Oases or Cufflinks. Novel 5′UTR extensions were validated by the presence of a H3K4me3 modification peak within a 2 kb region from the novel 5′end of the transcript. Novel 3′UTR extensions were validated by the presence of sequence reads corresponding to transcripts’ polyA tails within a 2 kb region from the novel 3′end of the transcript. A detailed description of this procedure is listed in Additional file 3: Supplementary Methods.

### General properties of novel transcript contigs

The expression levels of novel transcript contigs were quantified as Reads Per Kilobase per Million of the total mapped RNA-seq reads (RPKM). The coding potential of novel transcript contigs was estimated using the CPC algorithm [23]. The presence of canonical donor and acceptor site splice signals within novel transcript contigs was identified using GT-AG motifs. Exon conservation was estimated using phastCons17way based on 17 vertebrate species’ genomes data from UCSC [50]. For each exon, we used the average of all nucleotides’ phastCon scores to represent its conservation. We further required more than 80% of exon’s nucleotides to have a valid phastCon score. The same sequence conservation calculation procedure was used for another two genome sequence categories: (i) randomly selected intergenic regions and (ii) annotated lncRNAs. The tissue specificity of transcript expression was estimated using RNA-seq data from Human Body map [4]. To increase tissue coverage, two deep sequencing datasets with comparable sequencing coverage (fetal brain and fetal liver [51]) were combined with Human Body map data, resulting in sequencing data from a total of 19 human tissues. All novel transcript contigs, with a mean expression >0.1 RPKM across tested tissues, were classified into three categories: (i) tissue-specific, (ii) tissue-selective and (iii) ubiquitously expressed. Detailed classification method description is listed in Additional file 3: Supplementary Methods. The nuclear and cytoplasmic localization preference of novel transcripts was estimated using RNA-Seq data from SK-N-SH cells (human neuroblastoma cell line, GSE30567) from ENCODE/Cold Spring Harbor labs. To analyze temporal expression patterns of novel transcripts in human PFC development, novel transcript expression levels were quantified separately in each of 14 human PFC samples with different ages. Age-related novel lncRNAs were identified using a polynomial regression-based age test [52] at *p* < 0.01 under FDR 2%. The p-value cutoff and corresponding FDR was calculated by 1,000 permutations of sample age labels. Detailed description of FDR estimation procedure is listed in Additional file 3: Supplementary Methods. The K-means clustering algorithm was used to classify age-related novel and annotated transcripts into 12 clusters. Within each cluster, Fisher’s exact test was used to calculate the enrichment of novel transcripts compared to all age-related novel and annotated transcripts. Fisher’s exact test *p* < 0.05 after Bonferroni correction was considered as significant.

### Expression correlation of sense/antisense gene pairs

Sense/antisense gene pairs were defined based on three types of overlapping scenarios: (1) annotated in Ensembl annotation; (2) just like the first type, except that the overlap comes from assembled contigs that went beyond Ensembl annotation; (3) splicing-based overlap supported by novel junction reads. More specifically: in (1), annotated overlap was identified by searching overlapped genes from different strands within Ensembl annotation; in (2) if an assembled contig, representing genuine extension of one gene (at 5′ or 3′ end), overlapped with another gene on different strand, it was considered as a sense/antisense pair. In many cases, type (2) represents further extension for overlapping gene pairs already annotated within Ensembl (Additional file 7: Table S4). In (3), novel junctions reads supporting overlapping gene pairs were identified by PalMapper [49] and Tophat [53] with default parameters. We only used novel junction reads that were supported by both algorithms, and further required that the junction reads should match annotated splice sites within one gene. Note that the three approaches used to define sense/antisense pairs are not mutually exclusive (Additional file 7: Table S4).

For every gene, we required a mean expression > =0.1 RPKM. Pairs involved in complex genomic loci, with more than two genes having the same overlapping pattern in multiple cases (tail-to-tail or head-to-head), were removed from further analysis.

Expression correlation for each overlapping sense/antisense gene pairs during postnatal development was measured by Pearson correlation coefficient, and non-overlapping gene pairs (closest in the genome in terms of location, and from different strands as for overlapping ones) were used as control to check the potential influence of antisense transcription. Further, the significance of this influence was assessed by sampling 100 times from the control in order to obtain the same number of gene pairs as overlapping ones.

### Novel transcript classification based on genomic context

Novel transcripts located outside of annotated gene regions were classified into four categories, based on their location with respect to the nearest annotated gene: upstream-sense, downstream-sense, upstream-antisense and downstream-antisense. The distance cutoff used to identify novel transcript – annotated gene pairs was defined using random transcript pairs distance distribution, calculated by 1,000 permutations of novel transcript loci along each chromosome (for each permutation, keeping the same number of novel transcripts on each strand of each chromosome). A detailed description of the cutoff selection procedure is listed in Additional file 3: Supplementary Methods. We used a Wilcoxon rank test to compare the observed distributions and each of the 200 simulated distributions of the correlation coefficients to determine how many of them pass the statistical significance cutoff. Specifically, for each permutation, we randomized the relationship between novel contig clusters and nearby protein-coding genes and estimated the statistical significance of the correlation distribution difference using Wilcoxon rank test. We found significantly stronger positive correlations for the actual data when compared to each of the 200 simulated distributions, for gene pairs composed of protein-coding genes and novel lncRNA in the upstream-sense, downstream-sense and upstream-antisense categories (Wilcoxon rank test, p < 0.05).

### Divergent transcription at promoters associated with upstream-antisense category

The divergent transcription from promoters was estimated by deepCAGE data from FANTOM4 [9]. Only deepCAGE data from brain tissues was used. To define the divergent transcription features specific to the promoters that were associated with novel transcripts from the upstream-antisense category, unidirectional and known bidirectional expressed annotated genes were used as background. The criteria to select unidirectional, known bidirectional promoters and novel bidirectional promoters were as follows: for known bidirectional promoters and novel bidirectional promoters, genes were required to form head-to-head gene pairs within the region of 2 kb from TSS. The choice of 2 kb as a distance cutoff to define bidirectional promoters in our study was dictated by artificial transcript shortening at the 5′-end as a result of the de novo assembly procedure. Specifically, the Illumina RNA-sequencing protocol used in our study includes a polyA enrichment procedure employing polyT primers. It results in a preferential coverage of the transcripts’ 3′ regions where the polyA tail is located leaving the 5′ part underrepresented and frequently incomplete, especially in cases of detectable RNA degradation. To assess the extent of this coverage bias, we tested distances between gene pairs, defined based on the de novo assembly results, for 745 known bidirectional promoters containing gene pairs annotated to be within 1 kb distance from one another. We found that for 184 (24.7%) of these 745 bidirectional promoters the distance was greater than 1 kb when based on de novo assembly results (Additional file 5: Figure S10b). Thus, when using 1 kb as a cutoff, close to 25% of the known bidirectional promoters will be missed. By contrast, when using a 2 kb distance cutoff, 91.8% of them are retained. Importantly, the false positive rate of the bidirectional promoter definition only increased to a total of 7.5% when changing from 1 kb to 2 kb distance cutoff. Using 1 kb instead of 2 kb cutoff did not alter results (Additional file 5: Figure S10). For unidirectional promoters, genes were required to have no annotated transcripts, or novel transcript contigs identified in this study, within the 5 kb region upstream of their TSS. The promoters defined as showing divergent transcription were required to have at least one CAGE tag on each strand. Unidirectional promoters were required to have at least two CAGE tags at the annotated gene’s strand, and zero tags at the opposite strand. The promoters containing no CAGE tags were excluded from analysis. Fisher’s exact test was used to calculate the divergent transcription feature enrichment.

### Properties of genes associated with novel upstream antisense transcripts

The protein-coding genes that showed significant positive correlation with the expression of upstream antisense lncRNAs (Pearson correlation *p* < 0.05 after Benjamini-Hochberg correction) were selected for functional feature analysis. Functional enrichment was conducted using a hypergeometric test implemented in the Genetrail package [54]. Functional terms with *p* < 0.05 after Benjamini-Hochberg correction were considered as significant. Protein-coding genes with mean expression >0.1RPKM in human PFC data were used as a background. Enriched GO terms were visualized after term redundancy reduction using REVIGO [32]. The same functional enrichment analysis procedure was applied to protein-coding genes associated with novel transcripts from the other three categories, as well as protein-coding genes associated with novel upstream antisense transcripts but not showing positive correlation.

H3K4me3 modification enrichment analysis between neurons and non-neuronal cells from human PFC was conducted using ChIPDiff [55]. H3K4me3 modification data from neurons and non-neuronal cells of human PFC was downloaded from [33]. The regions with more than two-fold higher H3K4me3 modification signals in neurons than in non-neuronal cells were considered as regions preferentially expressed in neurons (assigned with a “N” flag). The regions with opposite modification signal patterns were considered as regions preferentially expressed in non-neural cells (assigned with a “non-N” flag). The significance was assessed by 1,000 permutations of N and non-N flag labels.

The list of mouse genes with known cell-type-specific expression patterns was downloaded from [34]. Human orthologs were determined using Biomart from Ensembl [20]. Fisher’s exact test was used to test the enrichment significance, and *p* < 0.05 after Bonferroni correction was considered as significant.

### Analysis of the DNA sequence and epigenetic features of uni-, known and novel bidirectional promoters

Uni-, known and novel bidirectional promoters were defined as described above. Three DNA sequence features (GC content, regulatory potential, sequence conservation) and two epigenetic features (H3K4me3 modification profile and DNA methylation status) were explored.

Specifically, GC content was measured as the G + C percentage of the promoter region. Regulatory potential was estimated using the Regulatory Potential (RP) Scores downloaded from UCSC [50]. RP Scores are a computational tool to aid in the identification of putative regulatory sites of the human genome [38]. For each promoter, we used the average RP score to represent its Regulatory Potential. We further required more than 80% of promoter nucleotides to have a valid RP score. Promoter region conservation was estimated using phastCon scores, based on 17 vertebrate species genome data and using the same approach as for the estimation of novel contig conservation. The differences with respect to each of the three DNA features among these three promoter types were tested using the Kolmogorov–Smirnov test.

H3K4me3 modification data from one adult human PFC was downloaded from [39]. H3K4me3 modification and input control data from rhesus macaque PFC was downloaded from [40]. H3K4me3 modification and input control data from Hela cells was downloaded from [41]. H3K4me3 modification density differences were tested using the Wilcoxon signed-rank test. For DNA methylation data, the DNA methylation status of the human PFC, measured by MeDIP sequencing (Methylated DNA Immunoprecipitation Sequencing), was downloaded from [39]. The DNA methylation level differences were tested using Wilcoxon signed-rank test.

The RNA-Seq data of PABPN1 knockdown and control experiments was downloaded from SRP015926 [48]. We adopted the same method used in [48] for expression quantification of known protein-coding genes, known lncRNAs and novel lncRNAs.

Additionally we compared the DNA sequence composition and epigenetic features of NBiPs and lncKBiPs that are formed by protein-code genes and known lncRNA pairs. Except for the shape of the H3K4me3 modification profile, significant differences for the other features can also be detected for these two promoter types. At the same time, the sequence and epigenetic properties of lncKBiPs more closely resemble known bidirectional promoters that are formed by pairs of protein-coding genes (pcKBiPs). Besides the sequence and epigenetic features, the effect of a PABPN1 knockdown on lncRNA associated with NBiPs was different compared to the effect seen for the known lncRNA and protein-coding genes that are associated with lncKBiPs and pcKBiPs (Additional file 5: Figure S8).

### Enriched transcription factor binding site detection in novel bidirectional promoter (NBiP) regions

Transcription factor binding sites (TFBSs) located within NBiP and KBiP regions were predicted using the MATCH algorithm based on TRANSFAC Release 11 [56]. To minimize false positive matches, the matrix file vertebrate_non_redundant_minFP.prf was used for TFBS prediction. Enriched TFBS in NBiP regions were identified by Fisher’s exact test, using KBiP regions as a background. Significantly enriched TFBS had to satisfy the following criteria: a) Benjamini-Hochberg adjusted p-value < 0.05; b) Fisher’s exact test odds ratio > 1.3. The background distribution of TFBS along NBiP was estimated by dinucleotide shuffle of NBiP sequences. Specifically, NBiP sequences were subjected to dinucleotide shuffle 1,000 times and the MATCH algorithm was applied to the shuffled sequences. CoCiter [37] was used to check the significance of association between transcription factors enriched in NBiP and KBiP, with the terms “neuron” and “neural”, respectively.

## Electronic supplementary material

Additional file 1: Table S1: Containing sample information. (DOC 44 KB)

Additional file 2: Table S2: Containing RNA-seq data mapping statistics. (DOC 42 KB)

Additional file 3: **Supplementary methods.** This file contains additional detailed methods description. (DOCX 61 KB)

Additional file 4: **Supplementary data 1 containing three tables.** **Table 1.** Contains information about all novel transcripts identified in this study. **Table 2.** Contains a list of 273 novel transcripts/protein-coding genes pairs originating at NBiPs and showing significantly positive expression correlation in PFC development. **Table 3.** Contains NBiPs identified in this study. (XLSX 3 MB)

Additional file 5: **Figure S1.** Shows major expression patterns of protein-coding genes and novel lncRNAs measured across human postnatal PFC development. **Figure S2.** Shows relative position and count distribution of assembled antisense transcripts within the sense region of annotated protein-coding genes. **Figure S3.** Shows expression correlation across postnatal PFC development of overlapping tail-to-tail sense/antisense gene pairs from different types of overlapping scenarios. **Figure S4.** Shows expression correlation across postnatal PFC development of overlapping head-to-head sense/antisense gene pairs from different types of overlapping scenarios. **Figure S5.** Shows divergent transcription at different promoter types. **Figure S6.** Shows H3K4me3 modification profiles at three promoter types. **Figure S7.** Shows H3K4me3 input/control data profiles at three promoter types. **Figure S8.** Shows the sequence and epigenetic features of four promoter types, including bidirectional promoters that are formed by known lncRNA and protein-coding gene pairs. **Figure S9.** Shows the correlation between the expression of protein-coding genes located on the sense and antisense strands. **Figure S10.** Shows the sequence and epigenetic features of three promoter types at a 1 kb distance cutoff. (DOC 3 MB)

Additional file 6: Table S3: Shows novel lncRNAs and known protein-coding gene enrichment in 12 clusters. (DOC 39 KB)

Additional file 7: Table S4: Shows number of sense/antisense gene pairs based on different annotation sources. (DOC 49 KB)

Additional file 8: Table S5: Shows CAGE tag distribution on forward and reverse strands for three promoter types. (DOC 34 KB)

Additional file 9: Table S6: Contains 3 tables. **Table 1.** Shows GO functions enriched in genes associated with novel upstream antisense lncRNAs based on Trinity assembly. **Table 2.** Shows GO functions enriched in genes associated with novel upstream antisense lncRNAs based on Cufflinks assembly. **Table 3.** Shows GO functions enriched in genes associated with novel upstream antisense lncRNAs based on intersection of Trinity and Cufflinks assembly. (XLS 50 KB)

Additional file 10: Table S7: Contains 2 tables. **Table 1.** Lists over-represented GO functions for genes associated with KBiPs. **Table 2.** Lists under-represented GO functions for genes associated with KBiPs. (XLS 51 KB)

Additional file 11: Table S8: Contains 2 tables. **Table 1.** Lists over-represented GO functions for genes associated with UniPs. **Table 2.** Lists under-represented GO functions for genes associated with UniPs. (XLS 38 KB)

Additional file 12: Table S9: Lists transcription factors showing binding site overrepresentation in NBiP. (XLS 24 KB)

Additional file 13: Table S10: Lists Transcription factors showing binding site overrepresentation in KBiPs. (XLS 22 KB)

Additional file 14: Table S11: Shows strand distribution of uniquely mapped reads. (DOC 42 KB)

Additional file 15: Table S12: Shows RNA-seq coverage at splice site sequences corresponding to sense/antisense splice junctions. (DOC 34 KB)

## Abbreviations

lncRNAs: Long non-coding RNAs
GO: Gene ontology
PFC: Prefrontal cortex
UTR: Untranslated region
TSS: Transcriptional start site
RPKM: Reads per kilobase per million reads
RP: Regulatory potential
NBiPs: Novel bidirectional promoters
KBiPs: Known bidirectional promoters
UniPs: Unidirectional promoters
TF: Transcription factor
TFBS: Transcription factor binding site.
