# Supplementary material for: Deep sequencing reveals a novel class of bidirectional promoters associated with neuronal genes
Source: BMC Genomics. 2014 Jun 10;15(1):457. doi: 10.1186/1471-2164-15-457 (PMC4094773; doi:10.1186/1471-2164-15-457)
Supplement: Supplementary file 1 — Additional file 1: Table S1: Containing sample information. (DOC 44 KB) [file 12864_2013_6226_MOESM1_ESM.doc]

# Additional file 1: Table S1. Sample information, adopted from Mazin et al. 2013^a^.

| **Sample index** | **Age (days)** | **Tissue** | **Accession number** | **Total Reads** |
| --- | --- | --- | --- | --- |
| S1 | 2 | Prefrontal cortex | SRR107727 | 21,277,649 |
| S2 | 4 | Prefrontal cortex | SRR111895 | 21,284,713 |
| S3 | 19 | Prefrontal cortex | SRR111896 | 20,754,409 |
| S4 | 34 | Prefrontal cortex | SRR111897 | 23,722,421 |
| S5 | 94 | Prefrontal cortex | SRR111898 | 23,416,250 |
| S6 | 204 | Prefrontal cortex | SRR111899 | 22,698,303 |
| S7 | 443 | Prefrontal cortex | SRR111900 | 23,934,412 |
| S8 | 787 | Prefrontal cortex | SRR111901 | 17,759,057 |
| S9 | 5,105 | Prefrontal cortex | SRR111902 | 19,901,399 |
| S10 | 9,277 | Prefrontal cortex | SRR111903 | 23,201,284 |
| S11 | 19,457 | Prefrontal cortex | SRR111904 | 16,019,209 |
| S12 | 24,090 | Prefrontal cortex | SRR111905 | 20,948,595 |
| S13 | 32,120 | Prefrontal cortex | SRR111906 | 21,032,459 |
| S14 | 35,770 | Prefrontal cortex | SRR111907 | 20,255,260 |
| **Total** | -- | -- | -- | 296,205,420 |

^a^ This table is adopted from Mazin et al. Molecular Systems Biology, 2013. The RNA-seq data were downloaded from SRA under accession number SRP005169.
