# Supplementary material for: Deep sequencing reveals a novel class of bidirectional promoters associated with neuronal genes
Source: BMC Genomics. 2014 Jun 10;15(1):457. doi: 10.1186/1471-2164-15-457 (PMC4094773; doi:10.1186/1471-2164-15-457)
Supplement: Supplementary file 2 — Additional file 2: Table S2: Containing RNA-seq data mapping statistics. (DOC 42 KB) [file 12864_2013_6226_MOESM2_ESM.doc]

**Additional file 2: Table S2. RNA-seq statistics**.

| **Sample** | **Index** | **Brain Region** | **Total reads** | **Uniquely mapped reads** | **Unique %** |
| --- | --- | --- | --- | --- | --- |
| 2 days | S1 | PFC | 21,277,649 | 13,092,207 | 61.53% |
| 4 days | S2 | PFC | 21,284,713 | 16,705,998 | 78.49% |
| 19 days | S3 | PFC | 20,754,409 | 15,209,444 | 73.28% |
| 34 days | S4 | PFC | 23,722,421 | 18,591,846 | 78.37% |
| 94 days | S5 | PFC | 23,416,250 | 17,700,518 | 75.59% |
| 204 days | S6 | PFC | 22,698,303 | 17,364,904 | 76.50% |
| 443 days | S7 | PFC | 23,934,412 | 18,412,452 | 76.93% |
| 787 days | S8 | PFC | 17,759,057 | 13,313,573 | 74.97% |
| 5105 days | S9 | PFC | 19,901,399 | 14,250,786 | 71.61% |
| 9277 days | S10 | PFC | 23,201,284 | 17,707,721 | 76.32% |
| 19457 days | S11 | PFC | 16,019,209 | 11,621,426 | 72.55% |
| 24090 days | S12 | PFC | 20,948,595 | 16,053,838 | 76.63% |
| 32120 days | S13 | PFC | 21,032,459 | 15,946,247 | 75.82% |
| 35770 days | S14 | PFC | 20,255,260 | 15,630,850 | 77.17% |
| **Total** | **--** | **--** | **296,205,420** | **221,601,810** | **74.81%** |
