# Supplementary material for: Deep sequencing reveals a novel class of bidirectional promoters associated with neuronal genes
Source: BMC Genomics. 2014 Jun 10;15(1):457. doi: 10.1186/1471-2164-15-457 (PMC4094773; doi:10.1186/1471-2164-15-457)
Supplement: Supplementary file 3 — Additional file 3: Supplementary methods. This file contains additional detailed methods description. (DOCX 61 KB) [file 12864_2013_6226_MOESM3_ESM.docx]

**Additional file 3: Supplementary Methods**

*Validation of novel elements of known annotation*

*1) Novel internal exons, novel splicing donor/acceptor splicing sites validation*

The assembly results obtained using two additional transcript assembly algorithms, Oases[[1](#_ENREF_1)] and Cufflinks[[2](#_ENREF_2)], were used to validate novel internal exons and novel splicing donor & acceptor splicing sites assembled by the Trinity algorithm. Similar to Trinity, Oases is a reference-free *de novo* assembly tool using de Bruijn graphs to assemble transcripts. The parameters to run Oases were as follows: for velveth (-k 25 –short -strand_specific) and for velvetg (-read_trkg yes -min_contig_lgth 300). After merging the overlapped transcript contigs, Oases reported a total of 38,715 aligned transcript contig clusters. Cufflinks is a reference-based assembly tool that constructs a parsimonious set of transcripts by reducing the comparative assembly problem to a problem in maximum matching in bipartite graphs. Cufflinks required mapped RNA-seq reads data as an input. For the mapping step, Tophat was used to map raw RNA-seq reads to the human genome (hg19) with the following parameters (-a 6 -m 2 --solexa1.3-quals -p 40 --no-coverage-search --microexon-search --segment-mismatches 3 --segment-length 25). Since our RNA-seq reads are strand-specific, the output mapping result of Tophat was revised to include strand information for each reads by adding the “XS” tag. These revised mapping results were used as an input for Cufflinks. The parameters to run Cufflinks were as follows (-p 40 --library-type fr-secondstrand --min-frags-per-transfrag 3). Cufflinks reported a total of 195,353 transcript contigs with a length longer than 300 nt. After merging overlapped contigs, 133,761 transcript contig clusters were obtained.

Novel internal exons and novel splicing donor/acceptor splicing sites identified using Trinity-based assembled transcripts were considered validated if they were found in Oases and/or Cufflinks assembly results. Similarly, validation of novel splicing donor & acceptor splicing sites required that the novel splicing sites were detected using Oases and/or Cufflinks.

*2) Novel 5’UTR extensions validation by the presence of H3K4me3 modification peaks*

Novel 5’UTR extensions were validated by the presence of H3K4me3 modification peaks within a 2 kb region from the 5’end. H3K4me3 modification data from human prefrontal cortex (PFC) was downloaded from [[3](#_ENREF_3)] and [[4](#_ENREF_4)]. BayesPeak algorithm [[5](#_ENREF_5)] was used to detect H3K4me3 modification peaks across the human genome (hg19). BayesPeak is a BioConductor package for the analysis of ChIP-seq experiment data. Its algorithm models the positions and orientations of the sequenced fragments and determines the locations of enriched areas, corresponding to binding sites and histone modifications, using a hidden Markov model and Bayesian statistical methodology.

The parameters to run BayesPeak were as follows: for each chromosome (iterations = 10000L, use.multicore = TRUE, mc.cores=5). The result reported by BayesPeak was further screened with a posterior probability cutoff of 0.95 for peak regions longer than 300 nt, and a posterior probability cutoff of 0.99 for the rest of the peaks. A novel 5’UTR extension was considered as validated if at least one H3K4me3 modification peak was detected within a 2 kb region from its 5’end. 1,000 simulations were used to test the significance of the validation. Specifically, for each simulation, the H3K4me3 modification peaks were randomly distributed across the human genome, preserving the length and number distribution on each strand for each chromosome based on actual data. The permutation-based p-value was calculated as the number of times of simulation that produced greater or equal number of verified peaks than observed in the actual data.

*3) Novel 3’UTR extension validation using sequence reads corresponding to transcripts’ polyA tails*

Novel 3’UTR extensions were validated using sequence reads corresponding to transcripts’ polyA tails, detected within a 2kb region from the 3’end. Sequence reads corresponding to transcripts’ polyA tails (polyA-end) from 14 human PFC samples were identified as follows: i) Raw reads from 14 human PFC samples were combined for mapping; unmapped reads were collected after Tophat mapping; ii) unmapped reads with more than 4As at the 3’end were retained for further analysis; iii) the putative polyA tail part of each unmapped sequence was trimmed one nucleotide each time and mapped to the human genome iteratively, the longest uniquely mapped part was retained for each sequence; iv) internal As artifacts were overcome by requiring the dissimilarity rate (D) between the sequence of putative polyA tails and corresponding genomic sequence to be larger than 0.6; v) nearby polyA-ends were merged if the distance between them was less than 32 nt, the middle point of merged region was considered as polyA-end position (the merge distance cutoff was determined by calculating the distance distribution between the nearest polyA-ends on the same strand). In total, 100,864 polyA-ends were obtained with 2,939,207 reads, with 60,186 (~60%) polyA-ends containing a canonical polyA adenylation signal (AAUAAA or AUUAAA) at the upstream 15-40 nt region. A novel 3’UTR extension was considered as validated if at least one polyA-end mapped within a 2kb region from the 3’end. We used 1,000 data simulations to test the significance of the validation. Specifically, for each simulation, all polyA-ends were randomly distributed across the human genome maintaining the actual number distribution on each strand of each chromosome. The permutation-based p-value was calculated as the number of times of simulation that produced a greater or equal number of verified 3’UTR extensions than that observed in the actual data.

*Quality evaluation of strand-specific sequencing*

To evaluate the accuracy of the strand specificity of our dataset, total reads were mapped to the human genome (hg19) using PalMapper (see following for detailed mapping procedure). On average 75% of reads mapped uniquely. We then checked the reads distribution along sense/antisense strands based on the protein-coding genes (PCG). PCGs comprised 94.1% of total reads mapped to transcripts annotated in Ensembl, and the average ratio of sense-to-antisense reads was ~50. We note that within the Ensembl annotation, there exist a substantial number of overlapping genes, located either on the same strand or on different strands. To avoid erroneous counting of sense and antisense reads, reads from overlapped regions on the same strands were counted only once, while reads from overlapped regions located on different strands were excluded.

To test whether mapped antisense reads could be caused by technical artifacts, we first measured the expression correlation reads coverage at sense and antisense strands of annotated PCGs. We predicted that the coverage of antisense artifacts should be proportional to the actual abundance of sense product. We found no significant correlation between sense and antisense gene coverage in any of the 14 samples. Secondly, we examined the sequence of reads crossing splicing junctions, as they have built-in directionality due to the asymmetry of consensus splice sites. The results show that less than 0.1% of the junction reads may represent technical artifacts mapping to junctions at the antisense orientation. Therefore antisense reads found in this study mostly arose from *bona fide* transcription.

*Mapping by PalMapper*

Due to genetic variation among human individuals, we allowed variation to occur in the read alignments and new splice junction identification. The transcript variants and the new splice junctions were discovered by first aligning the RNA-seq reads to the genome. For this, we allowed up to four mismatches, two gaps and four edit operations (complete list of the parameters used is below). We filtered the variants and new splice junctions to obtain a confident set, and only took variants that were confirmed by at least five reads and at least 25% of the reads at that position. The splice junctions were retained if they were confirmed by at least three reads, if the length of the alignment was longer than 15 bp at each side of splice junction, and if the splice site was scored within the top 95% by a splice site detector trained on the annotated splice sites. We then used these transcript variants and new splice junctions in the final alignment.

The final alignment was performed with a slightly more stringent parameter setting to obtain a high quality alignment. We allowed up to four mismatches, one gap and four edit operations (see complete list of the parameters used below).

*Parameters used for human alignment in the first round:*

Reference Genome : hg19,GRch37

spliced_alignment="1"

stranded="1"

Mismatches: 4

Gaps: 2

Edit Operations: 4

Minimal seed length: 25

Miminal length of long hit: 35

Minimal length of short hit: 12

Mimimal combined length: 45

Longest intron length: 100000

Maximum number of introns in the alignment: 2

Maximum number of spliced alignments per read: 5

Distance to tolerate between hit and existing hit cluster 10

Number of alignments that leads to ignoring the read 1000

Report splice site with confidence more than 0.95

trigger spliced alignment, if unspliced alignment has at least 0 edit operations

trigger spliced alignment, if unspliced alignment has at least 0 gaps operations

Extension of the read region up- and downstream for triggering spliced alignments by presence of splice sites 5

Minimal exon length 12

number of matches required for identifying a splice site 10

Used a splice-site model trained on the gene annotation

Trim reads down to a length of 70 bp if no alignment is done

Trim the reads in steps of 3 if no alignment is found

Do not allow gaps in the last 10 bp

*Parameters used for human alignment in the second round:*

GFF=/fml/ag-raetsch/share/databases/genomes/H_sapiens/hg19_philipp/Homo_sapiens.GRCh37.62.gff3

spliced_alignment="1"

stranded="1"

Mismatches: 4

Gaps: 1

Edit Operations: 4

Minimal seed length: 20

Miminal length of long hit: 20

Minimal length of short hit: 12

Mimimal combined length: 45

Longest intron length: 20000

Maximum number of introns in the alignment: 2

Maximum number of spliced alignments per read: 5

Distance to tolerate between hit and existing hit cluster 10

Number of alignments that leads to ignoring the read 1000

Report splice site with confidence more than 0.95

trigger spliced alignment, if unspliced alignment has at least 0 edit operations

trigger spliced alignment, if unspliced alignment has at least 0 gaps operations

Extension of the read region up- and downstream for triggering spliced alignments by presence of splice sites 5

Minimal exon length 12

number of matches required for identifying a splice site 10

Used a splice-site model trained on the gene annotation

*Tissue expression specificity of novel transcript contigs*

The tissue expression specificity of novel transcript contigs was estimated using deep-sequencing data from 19 human tissues, including 17 human tissues from Human Body map [[6](#_ENREF_6)] and two RNA-seq-based datasets (fetal brain and fetal liver [[7](#_ENREF_7)]) with comparable sequence reads coverage. All novel transcript contigs with mean expression >=0.1 RPKM across tissues were classified into three categories: tissue-specific expression category, tissue-selective expression category and ubiquitous-expression category. The DPM score was used for classification [[8](#_ENREF_8)]. DPM is a statistical parameter introduced as a sensitive indicator in quantitative estimation of gene expression patterns [[8](#_ENREF_8)]. The classification procedure was carried out as follows:

i) We determined the DPM cutoff for the three-category classification based on protein-coding genes. To do this, we first built three data sets collected from previous publications, which include a ubiquitously expressed protein-coding gene set (UBGs), a tissue-specific expressed protein-coding gene set (TSpGs) and a tissue-selective expressed gene set (TSlGs). The UBGs was built by intersecting the three ubiquitously expressed gene lists collected from [[9-11](#_ENREF_9)]. TSpGs were collected from TissueDistributionDBs. The protein-coding genes excluded from the UBGs or TSpGs were considered as TSlGs. Next, DPM was calculated for each expressed protein-coding gene (mean RPKM >0.1 across 19 tissues). Based on the DPM distribution of UBGs, TSpGs and TSlGs, a DPM cutoff (0.7 for UBGs and TSlGs; 0.9 for TSpGs and TSlGs) was selected to balance the precision rate of UBGs classification (0.83) and TSpGs classification (0.82). Using this cutoff, expressed protein-coding genes were separated into 9,722 ubiquitously expressed genes (DPM<0.7), 4,497 tissue-selective expressed genes (0.7<=DPM<=0.9) and 4,440 tissue-specific expressed genes (DPM>0.9). To further estimate the sensitivity of such classification, we calculated the overlaps between our ubiquitously expressed genes and the ubiquitously expressed genes annotated as such in [[12](#_ENREF_12)]. The result showed that 6,274 (~89%) out of 7,056 ubiquitously expressed genes defined by [[12](#_ENREF_12)] were also identified as ubiquitous by our method.

ii) We applied the DPM cutoff, obtained based on protein-coding genes, to novel transcript contigs with expression >0.1 RPKM across human tissues. This resulted in the following transcript distribution across the three categories: ubiquitously expressed (3,253), tissue-selective expressed (15,890) and tissue-specific expressed (11,863).

*FDR estimation for age-related novel lncRNAs and protein-coding genes*

A polynomial regression-based age test developed in [[13](#_ENREF_13)] was used to identify age-related lncRNAs and protein-coding genes at *p*<0.01 under FDR 2% (*q*<0.02). The p-value cutoff and corresponding q-value was calculated by 1,000 permutations of age labels. Specifically, all expressed lncRNAs and coding-genes (average RPKM>0.1) from 14 human PFC samples were summarized into one data matrix (each row represents the expression of one gene across 14 postnatal ages, each column represents all gene expression level from one of 14 postnatal ages). For each permutation, the 14 columns corresponding to the 14 ages were shuffled resulting in one simulated data matrix, and the age test was further applied to this simulated data matrix to obtain age related genes. At a chosen nominal p-value cutoff, the q-value was calculated as the ratio of the median value of number of age-related genes, obtained by 1,000 times permutation, to the number of age-related genes in the actual data.

*Novel lncRNAs classification based on genomic context*

Novel lncRNA outside of annotated known gene regions were classified into four categories based on their location with respect to the nearest annotated protein-coding genes: upstream-sense, downstream-sense, upstream-antisense and downstream-antisense. The distance cutoff used to define novel transcript – annotated gene pairs was chosen using a random transcript pairs distance distribution calculated by 1,000 permutations of novel transcript loci along each chromosome. Specifically, for each permutation, all novel lncRNAs located outside of annotated known gene regions were randomly distributed across the human genome maintaining the novel lncRNA number and length distribution on each strand of each chromosome in accordance to the actual data. The distance between each simulated lncRNA and the nearest known gene partner was further calculated and used to estimate random transcript pairs distance distribution. Based on 1,000 permutations, the distance cutoff under 5% FDR was chosen by comparing the distance distribution of the actual data with the distribution of random transcript pairs distances.

*Properties of genes associated with novel upstream antisense lncRNA assembly using Cufflinks*

Of all transcripts determined based on cufflinks assembly, 493 protein-coding genes showing a significantly positive correlation with expression of upstream antisense lncRNA were selected based on Pearson correlation (*p*<0.05 after Benjamini-Hochberg correction). Functional enrichment analysis was performed using the hypergeometric test implemented in Genetrail. Only functional terms with *p*<0.05 after Benjamini-Hochberg correction were considered as significant. The neurons and non-neuronal H3K4me3 modification enrichment analysis, and the cell type specific gene enrichment analysis, were carried out using the same procedure for a set of 273 Trinity-based novel-upstream antisense lncRNA and gene pairs defined in the main text. The result of the H3K4me3 modification enrichment analysis showed that H3K4me3 modification signals are significantly higher in neuron than in non-neuronal cells for 493 protein-coding genes (Simulation test, *p*<10^-9^). For cell type specific gene enrichment analysis, the results showed that 493 protein-coding genes are enriched in neuron-specific expressed genes (*p*<0.0005 after Bonferroni correction), but not for astrocyte-specific (*p*>0.1 after Bonferroni correction) and oligodendrocyte-specific expressed genes (p>0.3, after Bonferroni correction).

**References**

1. Schulz MH, Zerbino DR, Vingron M, Birney E: **Oases: robust de novo RNA-seq assembly across the dynamic range of expression levels.** *Bioinformatics* 2012, **28:**1086-1092.

2. Trapnell C, Williams BA, Pertea G, Mortazavi A, Kwan G, van Baren MJ, Salzberg SL, Wold BJ, Pachter L: **Transcript assembly and quantification by RNA-Seq reveals unannotated transcripts and isoform switching during cell differentiation.** *Nat Biotechnol* 2010, **28:**511-515.

3. Maunakea AK, Nagarajan RP, Bilenky M, Ballinger TJ, D'Souza C, Fouse SD, Johnson BE, Hong C, Nielsen C, Zhao Y, et al: **Conserved role of intragenic DNA methylation in regulating alternative promoters.** *Nature* 2010, **466:**253-257.

4. Cheung I, Shulha HP, Jiang Y, Matevossian A, Wang J, Weng Z, Akbarian S: **Developmental regulation and individual differences of neuronal H3K4me3 epigenomes in the prefrontal cortex.** *Proc Natl Acad Sci U S A* 2010, **107:**8824-8829.

5. Spyrou C, Stark R, Lynch AG, Tavare S: **BayesPeak: Bayesian analysis of ChIP-seq data.** *BMC Bioinformatics* 2009, **10:**299.

6. Cabili MN, Trapnell C, Goff L, Koziol M, Tazon-Vega B, Regev A, Rinn JL: **Integrative annotation of human large intergenic noncoding RNAs reveals global properties and specific subclasses.** *Genes Dev* 2011, **25:**1915-1927.

7. Ameur A, Zaghlool A, Halvardson J, Wetterbom A, Gyllensten U, Cavelier L, Feuk L: **Total RNA sequencing reveals nascent transcription and widespread co-transcriptional splicing in the human brain.** *Nat Struct Mol Biol* 2011, **18:**1435-1440.

8. Pan JB, Hu SC, Wang H, Zou Q, Ji ZL: **PaGeFinder: quantitative identification of spatiotemporal pattern genes.** *Bioinformatics* 2012, **28:**1544-1545.

9. Warrington JA, Nair A, Mahadevappa M, Tsyganskaya M: **Comparison of human adult and fetal expression and identification of 535 housekeeping/maintenance genes.** *Physiol Genomics* 2000, **2:**143-147.

10. Hsiao LL, Dangond F, Yoshida T, Hong R, Jensen RV, Misra J, Dillon W, Lee KF, Clark KE, Haverty P, et al: **A compendium of gene expression in normal human tissues.** *Physiol Genomics* 2001, **7:**97-104.

11. Eisenberg E, Levanon EY: **Human housekeeping genes are compact.** *Trends Genet* 2003, **19:**362-365.

12. Ramskold D, Wang ET, Burge CB, Sandberg R: **An abundance of ubiquitously expressed genes revealed by tissue transcriptome sequence data.** *PLoS Comput Biol* 2009, **5:**e1000598.

13. Somel M, Guo S, Fu N, Yan Z, Hu HY, Xu Y, Yuan Y, Ning Z, Hu Y, Menzel C, et al: **MicroRNA, mRNA, and protein expression link development and aging in human and macaque brain.** *Genome Res* 2010, **20:**1207-1218.
