# Supplementary material for: Deep sequencing reveals a novel class of bidirectional promoters associated with neuronal genes
Source: BMC Genomics. 2014 Jun 10;15(1):457. doi: 10.1186/1471-2164-15-457 (PMC4094773; doi:10.1186/1471-2164-15-457)
Supplement: Supplementary file 5 — Additional file 5: Figure S1. Shows major expression patterns of protein-coding genes and novel lncRNAs measured across human postnatal PFC development. Figure S2. Shows relative position and count distribution of assembled antisense transcripts within the sense region of annotated protein-coding genes. Figure S3. Shows expression correlation across postnatal PFC development of overlapping tail-to-tail sense/antisense gene pairs from different types of overlapping scenarios. Figure S4. Shows expression correlation across postnatal PFC development of overlapping head-to-head sense/antisense gene pairs from different types of overlapping scenarios. Figure S5. Shows divergent transcription at different promoter types. Figure S6. Shows H3K4me3 modification profiles at three promoter types. Figure S7. Shows H3K4me3 input/control data profiles at three promoter types. Figure S8. Shows the sequence and epigenetic features of four promoter types, including bidirectional promoters that are formed by known lncRNA and protein-coding gene pairs. Figure S9. Shows the correlation between the expression of protein-coding genes located on the sense and antisense strands. Figure S10. Shows the sequence and epigenetic features of three promoter types at a 1 kb distance cutoff. (DOC 3 MB) [file 12864_2013_6226_MOESM5_ESM.doc]

**Additional file 5**

**Figure S1.** Major expression patterns of protein-coding genes and novel lncRNA measured across human postnatal PFC development. The K-means algorithm was used to group expressed lncRNAs and protein-coding genes (mean RPKM>=0.1) into 12 clusters. Each panel shows the expression pattern in one of the gene clusters. The x-axis shows the age of individuals on a log2 day scale. The y-axis shows standardized (Z-transformed) expression levels, where each unit indicates one standard deviation difference from the mean. Numbers above each panel show numbers of novel lncRNAs and protein-coding transcripts in each cluster. Clusters significantly enriched in novel lncRNAs are shown in red, clusters enriched in protein-coding genes – in blue. Clusters showing no significant enrichment are shown in gray.


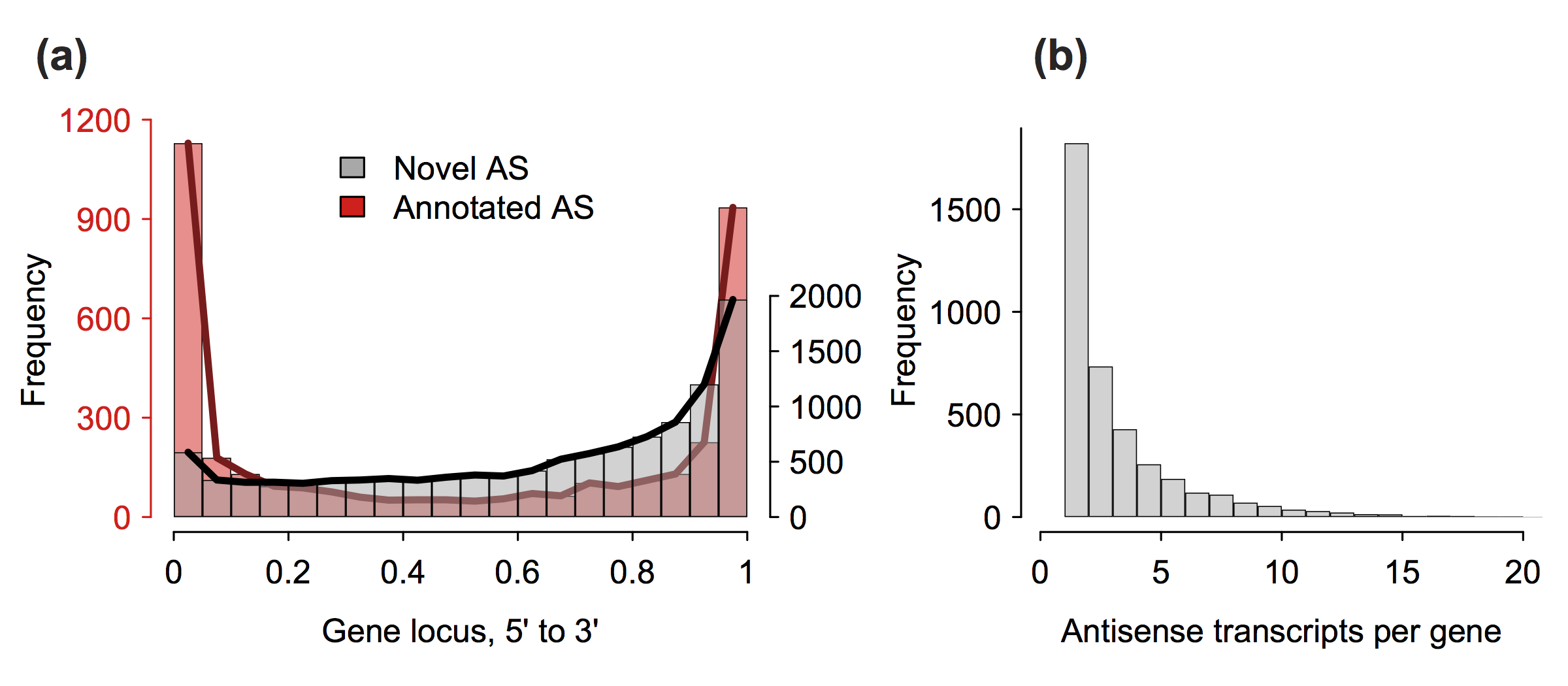


**Figure S2.** Relative position and count distribution of assembled antisense transcripts within the sense region of annotated protein-coding genes. (a) The relative position of novel antisense transcripts (black curve) and annotated antisense transcripts (red curve) within the sense region of annotated protein-coding genes. (b) The number of novel antisense transcripts within the sense region of annotated protein-coding genes.


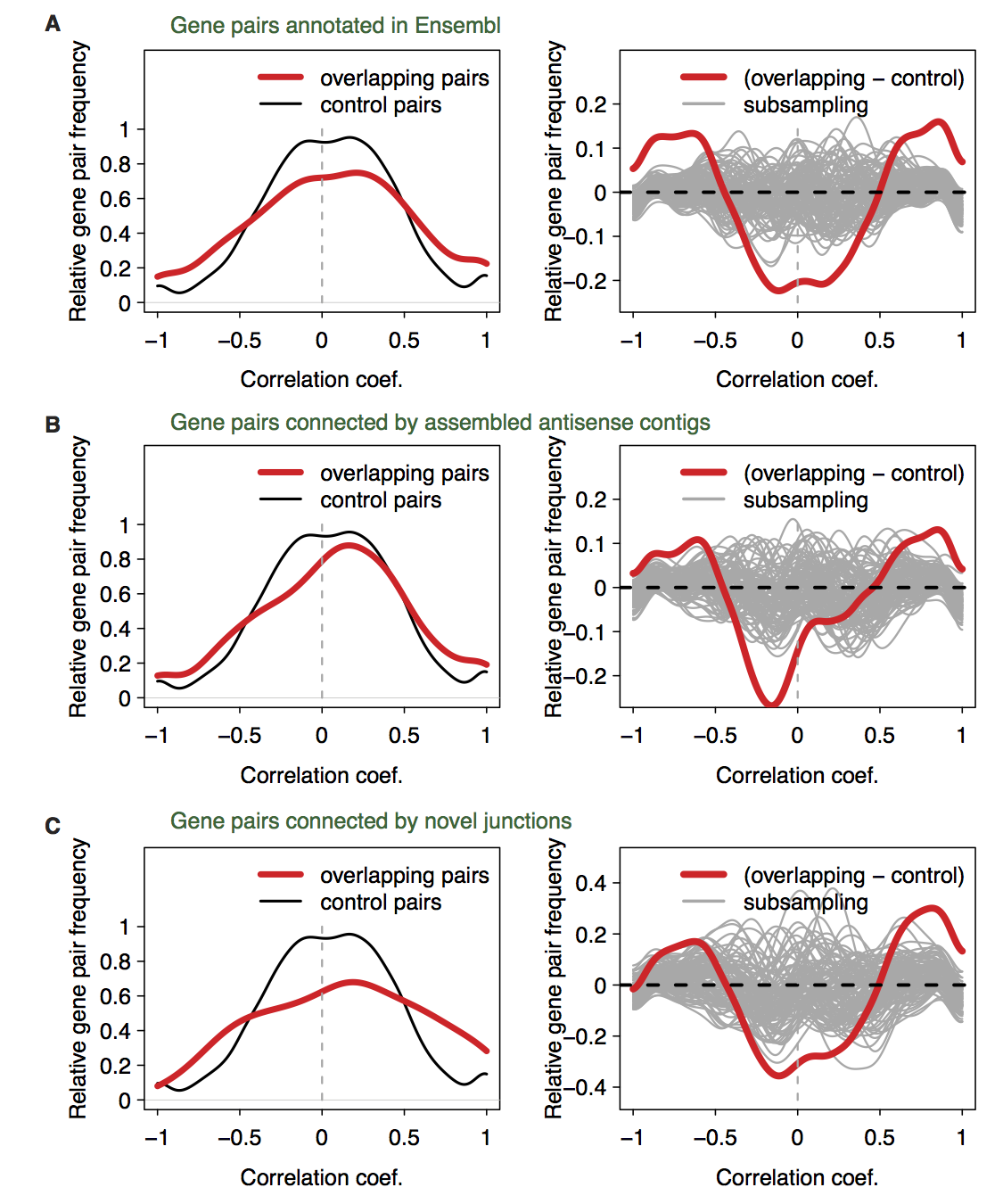


**Figure S3.** Expression correlation across postnatal PFC development of overlapping tail-to-tail sense/antisense gene pairs from different types of overlapping scenarios. The sense/antisense pairs were defined based on: (a) Ensembl annotation; (b) assembled contigs overlapping with annotated genes from different strands; (c) splicing-based overlap, supported by novel junction reads. Left panels: distribution of Pearson correlation coefficients calculated based on expression of tail-to-tail sense and antisense pairs (red) and non-overlapping control pairs (black) during postnatal PFC development. Right panels: Difference between the kernel density distribution of the overlapping tail-to-tail gene pairs’ correlation (red) and control pairs. The red line indicates the overlapping pairs, while the gray lines represent 100 simulations, generated by randomly subsampling the same number of control pairs as overlapping pairs.


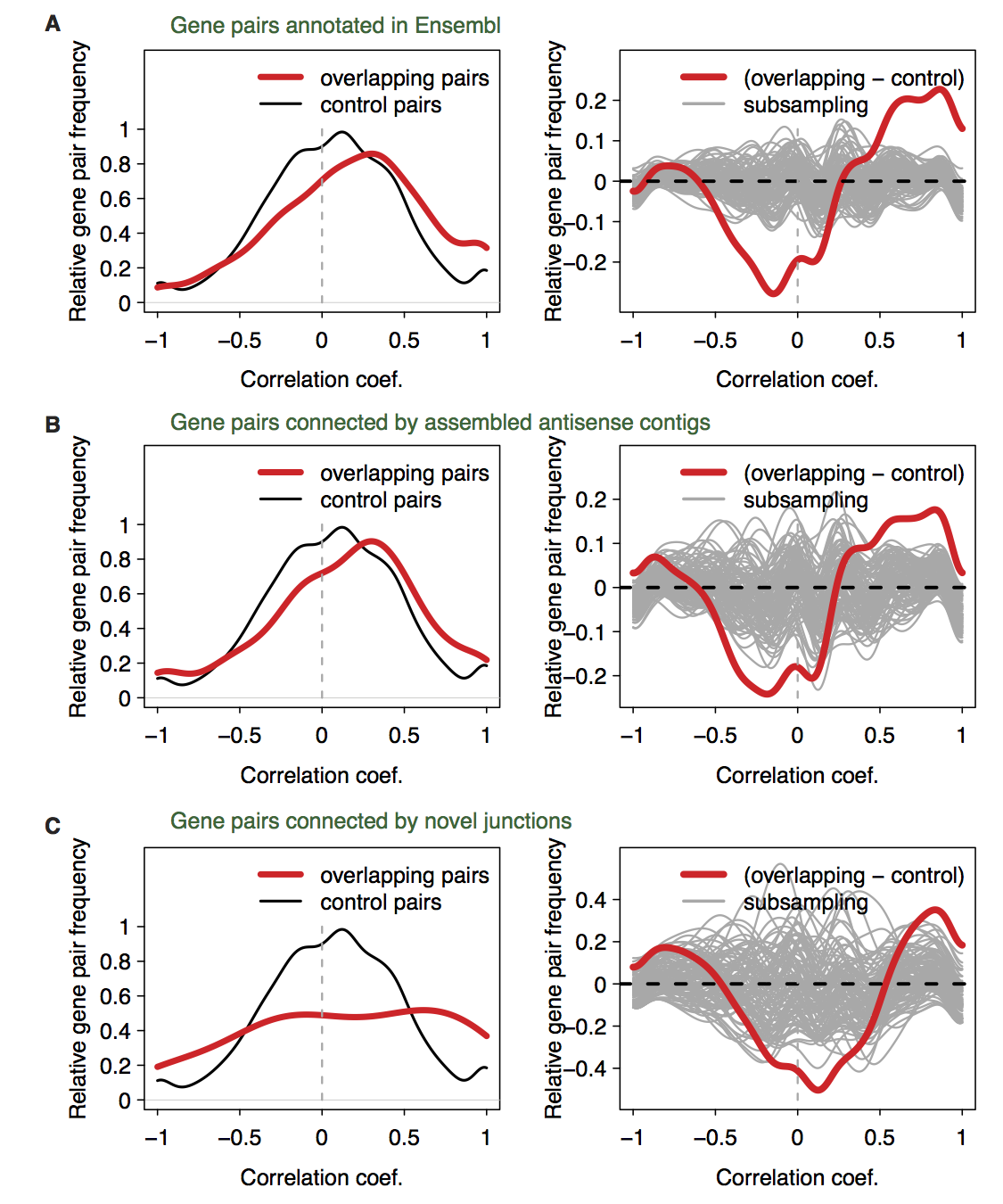


**Figure S4.** Expression correlation across postnatal PFC development of overlapping head-to-head sense/antisense gene pairs from different types of overlapping scenarios. The sense/antisense pairs were defined based on: (a) Ensembl annotation; (b) assembled contigs overlapping with annotated genes from different strands; (c) splicing-based overlap, supported by novel junction reads. Left panels: distribution of Pearson correlation coefficients calculated based on expression of head-to-head sense and antisense pairs (red) and non-overlapping control pairs (black) during postnatal PFC development. Right panels: Difference between the kernel density distribution of the overlapping head-to-head gene pairs’ correlation (red) and control pairs. The red line indicates the overlapping pairs, while the gray lines represent 100 simulations, generated by randomly subsampling the same number of control pairs as overlapping pairs.

**Figure S5.** Divergent transcription at different promoter types. Divergent transcription was measured using deepCAGE data from brain tissues. The y-axis shows the ratio between the number of promoters with bi- and uni-directional expression detected using deepCAGE data, for each promoter type: red – novel bidirectional promoters (NBiPs) with significantly positive expression correlation between novel lncRNA and mRNA expression (273-pairs); orange – all NBiPs; blue – unidirectional promoters (UniPs); white – known bidirectional promoters (KBiPs).


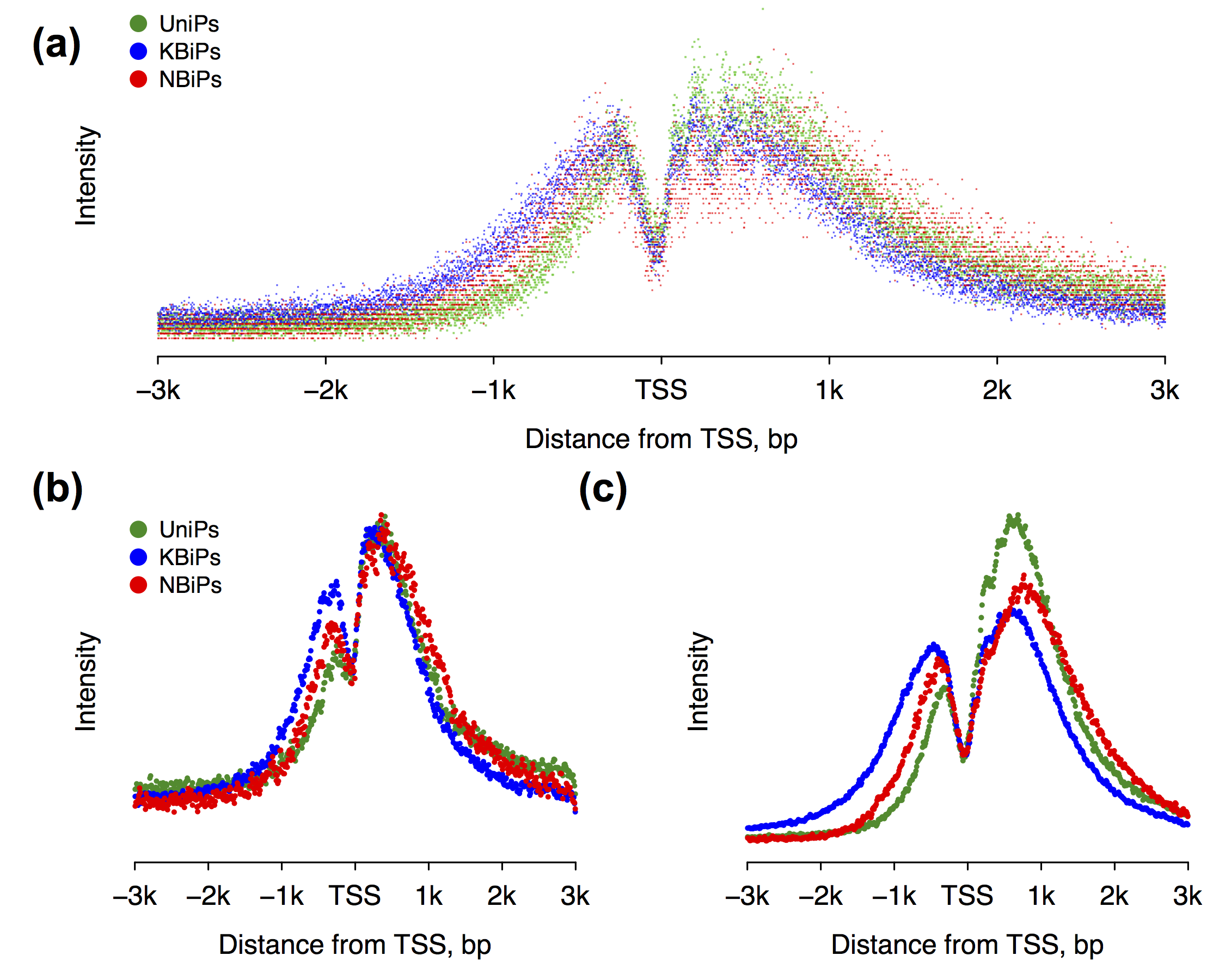


**Figure S6.** H3K4me3 modification profiles at three promoter types. The profiles are based on: (a) human PFC; (b) macaque PFC; (c) HeLa cells. Green – UniPs, blue – KBiPs; red – NBiPs.

**Figure S7.** H3K4me3 input/control data profiles at three promoter types. The profiles are based on: (a) human PFC; (b) macaque PFC; (c) HeLa cells. The left panels show the H3K4me3 modification profiles and the right panels show the H3K4me3 modification density at promoter regions. Green – UniPs, blue – KBiPs; red – NBiPs.


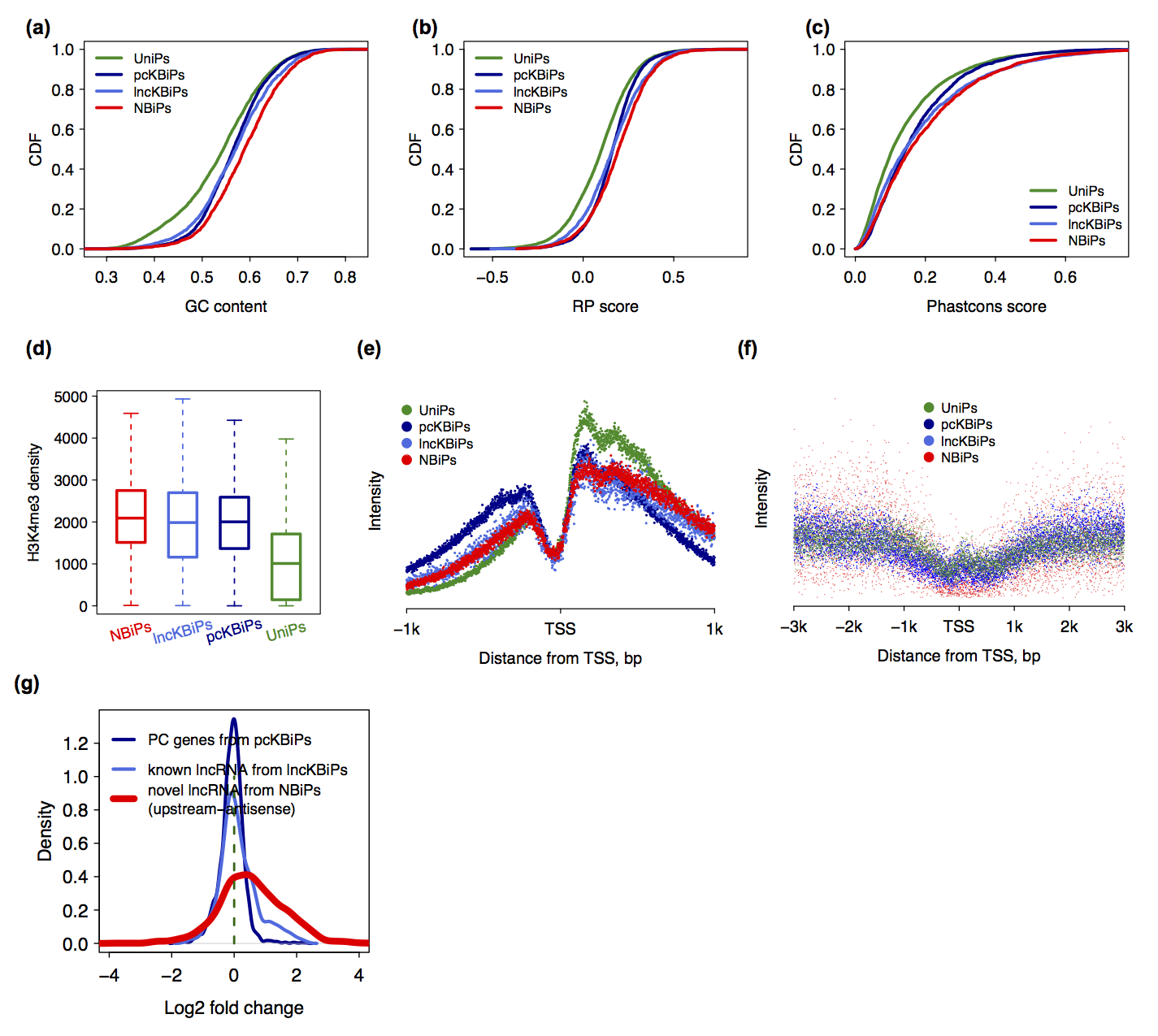


**Figure S8.** Sequence and epigenetic features of the four promoter types. The promoter types are marked as follows: unidirectional promoters – UniPs; bidirectional promoters formed by protein-code gene and known lncRNA pairs – lncKBiPs; bidirectional promoters formed by protein-code gene pairs – pcKBiPs; bidirectional promoters formed by protein-code gene and novel lncRNA pairs – NBiPs. (a), (b) and (c) The cumulative distributions of GC content, Regulatory Potential and sequence conservation for the three promoter types: UniPs (green), lncKBiPs (light blue), pcKBiPs (dark blue) and NBiPs (red). Promoter sequence conservation was calculated using phastCons scores, based on 17 vertebrate species’ genomes. Promoter Regulatory Potential was calculated using Regulatory Potential (RP) scores. (d), (e) The density (panel d) and the shape (panel e) of the H3K4me3 modification profiles at each promoter type. (f) DNA methylation profile at each promoter type. (g). The expression change distribution for different transcript types in a PABPN1 knockdown experiment. The positive values indicate expression upregulation following PABPN1 knockdown. Red: novel lncRNAs from NBiPs; light blue: known lncRNAs from lncKBiPs; dark blue: protein-coding genes from pcKBiPs.

**
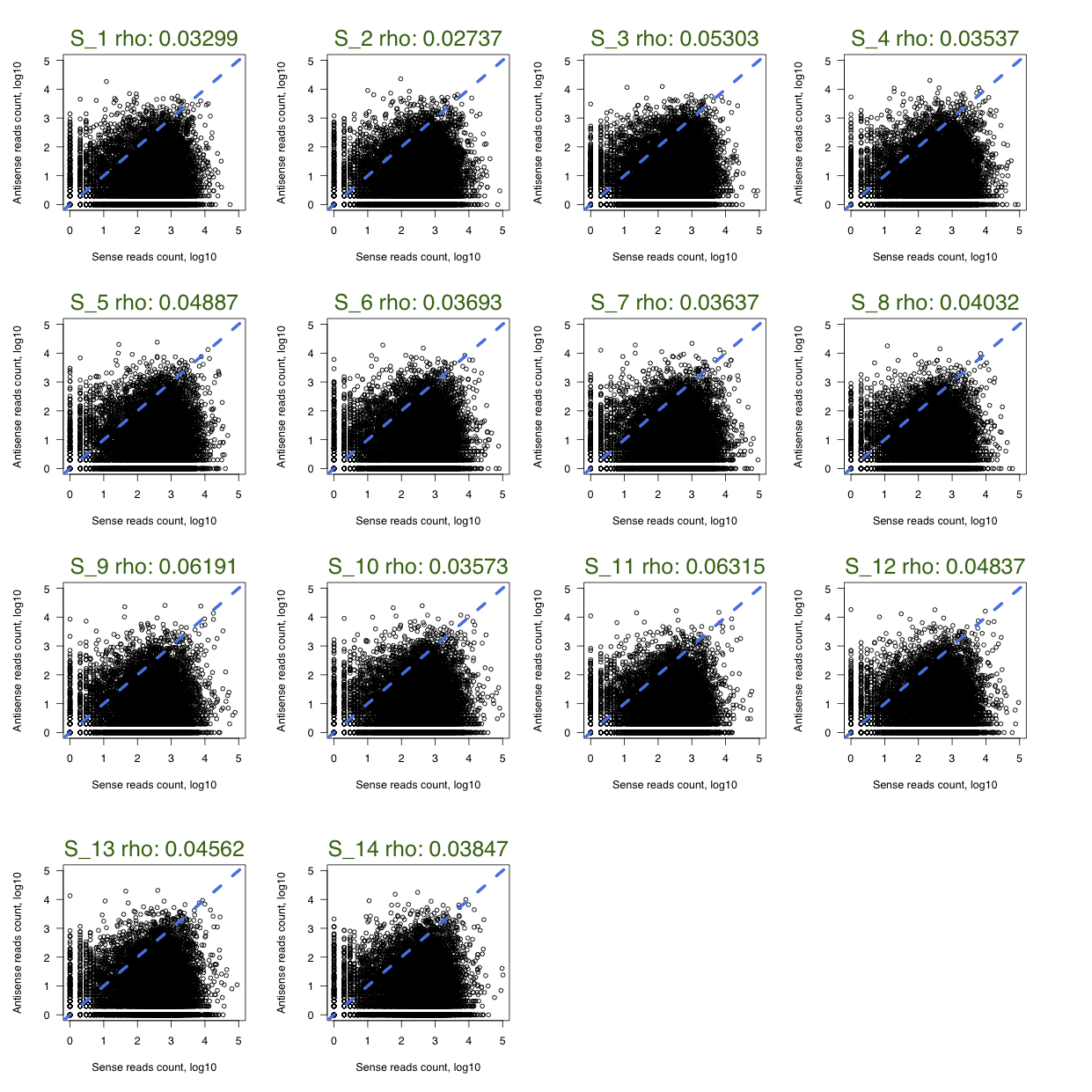
**

**Figure S9.** The correlation between expression of protein-coding genes located at sense and antisense strands. The x and y-axis represent the sense and antisense reads count (log10 scale) at protein-coding genes. The panel labels show sample index (from newborn to old individual) and Spearman’s correlation *rho* value.


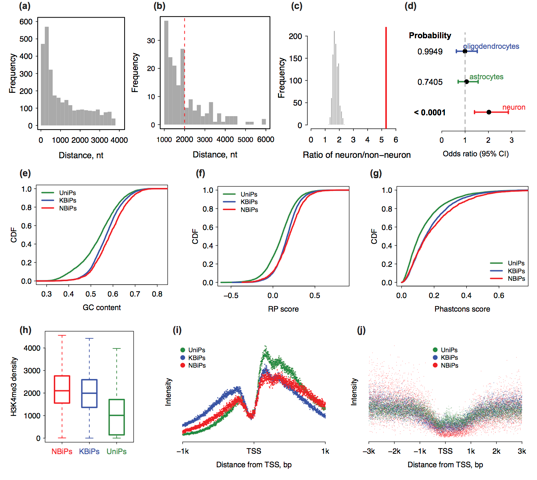


**Figure S10.** Genetic and epigenetic properties of the three promoter types at 1 kb bidirectional promoter definition cutoff. (a) The distribution of distances between novel lncRNA and the nearest protein-coding gene from the upstream antisense category. (b) The distance distribution for 184 known bidirectional promoters not recognized to pass a 1 kb distance cutoff based on the *de novo* assembly. For 123 of the 184 promoters the distance was within 2 kb (vertical dashed red line). (c) Expression specificity estimation based on H3K4me3 modification data from neurons and non-neural cells of human PFC. (d) Expression specificity estimation based on cell type specific expression data from mouse neocortex. (e), (f) and (g) The cumulative distributions of GC content, Regulatory Potential and sequence conservation for the three promoter types: UniPs (green), KBiPs (blue) and NBiPs (red). Promoter sequence conservation was calculated using PhastCons scores, based on 17 vertebrate species’ genomes. Promoter Regulatory Potential was calculated using Regulatory Potential (RP) scores. (h), (i) The density (panel h) and the shape (panel i) of H3K4me3 modification profiles at each of the three promoter types. (j) DNA methylation profile at each of the three promoter types. For abbreviation, UniPs: Unidirectional promoters; KBiPs: bidirectional promoters; NBiPs: bidirectional promoters formed by protein-code gene and novel lncRNA pairs.
