# Supplementary material for: Deep sequencing reveals a novel class of bidirectional promoters associated with neuronal genes
Source: BMC Genomics. 2014 Jun 10;15(1):457. doi: 10.1186/1471-2164-15-457 (PMC4094773; doi:10.1186/1471-2164-15-457)
Supplement: Supplementary file 6 — Additional file 6: Table S3: Shows novel lncRNAs and known protein-coding gene enrichment in 12 clusters. (DOC 39 KB) [file 12864_2013_6226_MOESM6_ESM.doc]

| **Additional file 6: Table S3. Novel lncRNAs and known protein-coding gene enrichment in 12 clusters** | | | | | | |  |
| --- | --- | --- | --- | --- | --- | --- | --- |
|  |  |  |  |  |  |  |  |
| **Cluster index** | **Novel lncRNAs** | **Annotated protein-coding genes** | **Enriched with novel lncRNAs** | **Enriched with protein-coding genes** | **Odds ratio** | **P-value** | **P-value after Bonferroni correction** |
| 1 | 474 | 1004 |  | * | 2.17 | 2.47E-42 | 2.96E-41 |
| 2 | 496 | 512 |  |  | 1.03 | 0.342088268 | 1 |
| 3 | 324 | 1504 |  | * | 5.36 | 9.21E-192 | 1.11E-190 |
| 4 | 1192 | 348 | * |  | 4.25 | 1.96E-134 | 2.35E-133 |
| 5 | 431 | 440 |  |  | 1.04 | 0.294408108 | 1 |
| 6 | 671 | 534 | * |  | 1.37 | 1.11E-07 | 1.33E-06 |
| 7 | 373 | 354 |  |  | 1.12 | 0.067230764 | 0.8067692 |
| 8 | 724 | 649 | * |  | 1.21 | 0.000566028 | 0.006792341 |
| 9 | 727 | 134 |  |  | 6.37 | 4.04E-114 | 4.85E-113 |
| 10 | 262 | 251 |  | * | 1.11 | 0.130335915 | 1 |
| 11 | 399 | 345 | * |  | 1.24 | 0.000109064 | 0.001308762 |
| 12 | 220 | 596 |  |  | 2.71 | 1.05E-38 | 1.26E-37 |
| **Total** | 6293 | 6671 |  |  |  |  |  |
