# Supplementary material for: Deep sequencing reveals a novel class of bidirectional promoters associated with neuronal genes
Source: BMC Genomics. 2014 Jun 10;15(1):457. doi: 10.1186/1471-2164-15-457 (PMC4094773; doi:10.1186/1471-2164-15-457)
Supplement: Supplementary file 7 — Additional file 7: Table S4: Shows number of sense/antisense gene pairs based on different annotation sources. (DOC 49 KB) [file 12864_2013_6226_MOESM7_ESM.doc]

# Additional file 7: Table S4. Number of sense/antisense gene pairs from different sources. ^a^

| Pairing types | Tail-to-tail | Head-to-head |
| --- | --- | --- |
| Annotated in Ensembl | 684 | 795 |
| Connected by assembled contigs | 886 (549^b^) | 527 (321^b^) |
| Connected by novel junctions | 136 (97^c^) | 49 (36^c^) |
| Sum | 1330^d^ | 1152^d^ |

# ^a^ for a detailed definition, please see Methods section.

# ^b^ Number in the parenthesis indicates novel gene pairs, calculated by removing those that already exist in the “Annotated in Ensembl” type.

^c^ Number in the parenthesis indicates novel gene pairs, calculated by removing those that already exist in the “Annotated in Ensembl” & “Connected by assembled contigs” types.

^d^ Redundant pairs from different sources were counted only once.
