# Supplementary material for: Deep sequencing reveals a novel class of bidirectional promoters associated with neuronal genes
Source: BMC Genomics. 2014 Jun 10;15(1):457. doi: 10.1186/1471-2164-15-457 (PMC4094773; doi:10.1186/1471-2164-15-457)
Supplement: Supplementary file 8 — Additional file 8: Table S5: Shows CAGE tag distribution on forward and reverse strands for three promoter types. (DOC 34 KB) [file 12864_2013_6226_MOESM8_ESM.doc]

| **Additional file 8: Table S5. CAGE tags distribution on forward and reverse strand of 3 types of promoters** | | | | |  |
| --- | --- | --- | --- | --- | --- |
|  |  |  |  |  |  |
| **Promoter type** | **Both strands have CAGE tag (bidirectional transcription)** | **Only one strand has CAGE tag (unidirectional transcription)** | **Bidirectional promoter percentage** | **Ratio of**  **Bidirectional transcription to unidirectional transcription** | **Divergent transcription feature enrichment *** |
| NBiPs | 1,311 | 453 | 0.7431973 | 2.89404 | 5.294206 |
| NBiPs with significantly positive correlation between lncRNA and protein-coding gene transcript | 201 | 61 | 0.7671756 | 3.295082 | 6.027171 |
| KBiPS | 2,460 | 740 | 0.76875 | 3.324324 | 6.08149 |
| UniPs | 1,587 | 2904 | 0.3533734 | 0.5464876 | − |
|  |  |  |  |  |  |
| * The divergent transcription feature enrichment was measured as the odds ratio of bidirectional and unidirectional transcription between other types of promoters with unidirectional promoters. Promoters with no CAGE tag were removed from this analysis. | | | | | |
|  | | |  |  |  |
