# Supplementary material for: Deep sequencing reveals a novel class of bidirectional promoters associated with neuronal genes
Source: BMC Genomics. 2014 Jun 10;15(1):457. doi: 10.1186/1471-2164-15-457 (PMC4094773; doi:10.1186/1471-2164-15-457)
Supplement: Supplementary file 14 — Additional file 14: Table S11: Shows strand distribution of uniquely mapped reads. (DOC 42 KB) [file 12864_2013_6226_MOESM14_ESM.doc]

**Additional file 14: Table S11**. Strand distribution of uniquely mapped reads.

| Sample | Index | All protein coding genes,  sense  strand | All protein coding genes,  antisense strand | S/AS* ratio | All protein coding genes’ exons,  sense strand | All protein coding genes’ exons,  antisense strand | S/AS ratio | All genes  sense  strand | All genes, antisense strand | S/AS ratio |
| --- | --- | --- | --- | --- | --- | --- | --- | --- | --- | --- |
| 2 days | S1 | 11152751 | 257409 | 43.33 | 9161662 | 62043 | 147.67 | 11786659 | 352143 | 33.47 |
| 4 days | S2 | 14389753 | 280110 | 51.37 | 12169930 | 72453 | 167.97 | 15220164 | 381535 | 39.89 |
| 19 days | S3 | 12693443 | 439512 | 28.88 | 10680086 | 97231 | 109.84 | 13485820 | 553732 | 24.35 |
| 34 days | S4 | 16129380 | 303634 | 53.12 | 14064225 | 85118 | 165.23 | 17028459 | 413335 | 41.20 |
| 94 days | S5 | 15148946 | 331225 | 45.74 | 13226444 | 98596 | 134.15 | 16071863 | 448467 | 35.84 |
| 204 days | S6 | 14981825 | 299333 | 50.05 | 13326425 | 81769 | 162.98 | 15825842 | 409612 | 38.64 |
| 443 days | S7 | 16020923 | 312777 | 51.22 | 14417087 | 94847 | 152.00 | 16886554 | 424934 | 39.74 |
| 787 days | S8 | 11437060 | 246521 | 46.39 | 10200984 | 74166 | 137.54 | 12124813 | 330335 | 36.70 |
| 5105 days | S9 | 12468583 | 258541 | 48.23 | 11492759 | 109446 | 105.01 | 13173734 | 343336 | 38.37 |
| 9277 days | S10 | 15641984 | 275940 | 56.69 | 14351696 | 87174 | 164.63 | 16446457 | 373150 | 44.07 |
| 19457 days | S11 | 10130579 | 214583 | 47.21 | 9251636 | 79409 | 116.51 | 10724378 | 285504 | 37.56 |
| 24090 days | S12 | 14092396 | 260334 | 54.13 | 12585115 | 86250 | 145.91 | 14857423 | 349501 | 42.51 |
| 32120 days | S13 | 13861279 | 288942 | 47.97 | 12198056 | 85437 | 142.77 | 14650220 | 384170 | 38.13 |
| 35770 days | S14 | 13662519 | 273954 | 49.87 | 12046601 | 78507 | 153.45 | 14437686 | 364428 | 39.62 |
| Total | -- | 191811421 | 4042815 | 47.45 | 169172706 | 1192446 | 141.87 | 202720072 | 5414182 | 37.44 |

* Number of sense reads divided by number of antisense reads
