# Supplementary material for: Deep sequencing reveals a novel class of bidirectional promoters associated with neuronal genes
Source: BMC Genomics. 2014 Jun 10;15(1):457. doi: 10.1186/1471-2164-15-457 (PMC4094773; doi:10.1186/1471-2164-15-457)
Supplement: Supplementary file 15 — Additional file 15: Table S12: Shows RNA-seq coverage at splice site sequences corresponding to sense/antisense splice junctions. (DOC 34 KB) [file 12864_2013_6226_MOESM15_ESM.doc]

**Additional file 15: Table S12. Splice site sequence RNA-seq coverage over sense/antisense junction sequences**.

| Sample Index | Canonical splice sites sequence  Sense* | Canonical splice site sequence  Antisense** | S/AS ratio | Percentage of canonical splice sites sequences |
| --- | --- | --- | --- | --- |
| S1 | 908383 | 125 | 7267.1 | 99.941% |
| S2 | 2050336 | 536 | 3825.3 | 99.930% |
| S3 | 1344138 | 224 | 6000.6 | 99.943% |
| S4 | 2517782 | 577 | 4363.6 | 99.933% |
| S5 | 1774802 | 623 | 2848.8 | 99.920% |
| S6 | 1924726 | 667 | 2885.6 | 99.916% |
| S7 | 2532259 | 317 | 7988.2 | 99.940% |
| S8 | 1648692 | 716 | 2302.6 | 99.910% |
| S9 | 1926370 | 747 | 2578.8 | 99.919% |
| S10 | 2469893 | 680 | 3632.2 | 99.927% |
| S11 | 1567187 | 872 | 1797.2 | 99.898% |
| S12 | 2256262 | 904 | 2495.9 | 99.914% |
| S13 | 2016208 | 951 | 2120.1 | 99.908% |
| S14 | 2064729 | 736 | 2805.3 | 99.920% |
| Sum | 27001767 | 8675 | 3112.6 | 99.923% |

* Canonical splice sites, Sense: GTAG, GCAG, ATAC

**Canonical splice sites, Antisense: CTAC, CTGC, GTAT
